# Estimation of the number of extreme pathways for metabolic networks

**DOI:** 10.1186/1471-2105-8-363

**Published:** 2007-09-27

**Authors:** Matthew Yeung, Ines Thiele, Bernard Ø Palsson

**Affiliations:** 1 Dept. of Bioengineering, University of California, San Diego, 9500 Gilman Drive, La Jolla, CA 92093-0412, USA; 2 Program in Bioinformatics, University of California, San Diego, 9500 Gilman Drive, La Jolla, CA 92093-0412, USA

## Abstract

**Background:**

The set of extreme pathways (ExPa), {**p**_*i*_}, defines the convex basis vectors used for the mathematical characterization of the null space of the stoichiometric matrix for biochemical reaction networks. ExPa analysis has been used for a number of studies to determine properties of metabolic networks as well as to obtain insight into their physiological and functional states *in silico*. However, the number of ExPas, *p *= |{**p**_*i*_}|, grows with the size and complexity of the network being studied, and this poses a computational challenge. For this study, we investigated the relationship between the number of extreme pathways and simple network properties.

**Results:**

We established an estimating function for the number of ExPas using these easily obtainable network measurements. In particular, it was found that log [*p*] had an exponential relationship with log⁡[∑i=1Rd−id+ici]
 MathType@MTEF@5@5@+=feaafiart1ev1aaatCvAUfKttLearuWrP9MDH5MBPbIqV92AaeXatLxBI9gBaebbnrfifHhDYfgasaacH8akY=wiFfYdH8Gipec8Eeeu0xXdbba9frFj0=OqFfea0dXdd9vqai=hGuQ8kuc9pgc9s8qqaq=dirpe0xb9q8qiLsFr0=vr0=vr0dc8meaabaqaciaacaGaaeqabaqabeGadaaakeaacyGGSbaBcqGGVbWBcqGGNbWzdaWadaqaamaaqadabaGaemizaq2aaSbaaSqaaiabgkHiTmaaBaaameaacqWGPbqAaeqaaaWcbeaakiabdsgaKnaaBaaaleaacqGHRaWkdaWgaaadbaGaemyAaKgabeaaaSqabaGccqWGJbWydaWgaaWcbaGaemyAaKgabeaaaeaacqWGPbqAcqGH9aqpcqaIXaqmaeaacqWGsbGua0GaeyyeIuoaaOGaay5waiaaw2faaaaa@4414@, where *R *= |*R*_*eff*_| is the number of active reactions in a network, d−i
 MathType@MTEF@5@5@+=feaafiart1ev1aaatCvAUfKttLearuWrP9MDH5MBPbIqV92AaeXatLxBI9gBaebbnrfifHhDYfgasaacH8akY=wiFfYdH8Gipec8Eeeu0xXdbba9frFj0=OqFfea0dXdd9vqai=hGuQ8kuc9pgc9s8qqaq=dirpe0xb9q8qiLsFr0=vr0=vr0dc8meaabaqaciaacaGaaeqabaqabeGadaaakeaacqWGKbazdaWgaaWcbaGaeyOeI0YaaSbaaWqaaiabdMgaPbqabaaaleqaaaaa@30A9@ and d+i
 MathType@MTEF@5@5@+=feaafiart1ev1aaatCvAUfKttLearuWrP9MDH5MBPbIqV92AaeXatLxBI9gBaebbnrfifHhDYfgasaacH8akY=wiFfYdH8Gipec8Eeeu0xXdbba9frFj0=OqFfea0dXdd9vqai=hGuQ8kuc9pgc9s8qqaq=dirpe0xb9q8qiLsFr0=vr0=vr0dc8meaabaqaciaacaGaaeqabaqabeGadaaakeaacqWGKbazdaWgaaWcbaGaey4kaSYaaSbaaWqaaiabdMgaPbqabaaaleqaaaaa@309E@ the incoming and outgoing degrees of the reactions *r*_*i *_∈ *R*_*eff*_, and *c*_*i *_the clustering coefficient for each active reaction.

**Conclusion:**

This relationship typically gave an estimate of the number of extreme pathways to within a factor of 10 of the true number. Such a function providing an estimate for the total number of ExPas for a given system will enable researchers to decide whether ExPas analysis is an appropriate investigative tool.

## Background

Extreme pathways (ExPas) of a metabolic network are the irreducible set of vectors that define the basis of the null-space of the network's stoichiometric matrix. Every allowable solution to the flux balance equations of a reaction network in steady state, **S**·*ν *= **0**, can be represented as a non-negative linear combination of the extreme pathway vectors. ExPas are biochemically and thermodynamically feasible pathways that transform a selection of the given substrates to a selection of allowable products. ExPas have been extensively used for the analyses of metabolic networks (see, for example, [1-5]). Typically, such analyses used ExPas to define possible phenotypic states of metabolic networks under different simulation conditions, to identify network redundancy, and to reveal *eigenpathways *that effectively characterize all relevant physiological states of a metabolic network. Modified versions of ExPa analyses have also been applied to regulatory networks [6,7] and signaling pathways [8]. Such applications are still in their infancy and are important research topics. However, as the size of a network increases, the redundancy of the network, that is, the number of different pathways that transform given substrate(s) to given product(s) [9,10], becomes more apparent, and the number of ExPas increases rapidly. Redundancy also exists in small systems but this can be easily handled and even provide insights to legitimate alternative pathways. As the number of ExPas increases at a drastic rate, performing insightful analyses using ExPas become increasingly difficult.

The fact that the set of ExPas of a biochemical reaction network defines the boundaries of its convex steady-state solution space makes them a valuable tool for metabolic network analysis. Further, they emphasize alternative pathways that exist in a network, which may otherwise be overlooked, and that can enrich the understanding of its possible physiological states. However, the increasing details included in reconstructed metabolic networks lead to the combinatorial explosion of the number of ExPas and their computation time. A method providing a good estimate for the final number of ExPas for a given system will enable researchers to decide whether ExPas analysis is a appropriate tool for their objectives.

Another method often used for characterizing the steady-state solution space for a biochemical reaction network is known as Elementary Modes (EMs) [11]. Both ExPa and EM analyses require the resulting solution vectors to be non-decomposable and unique. In addition, ExPa vectors are required to be systemically independent [12]. As a result, ExPas for a system are a minimal set of EMs, and the number of ExPas is less than or equal to the number of EMs. Since both ExPas and EMs are biochemically and thermodynamically feasible pathways, the number of these pathways cannot be estimated using traditional graph theoretical algorithms, such as the Dijkstra's algorithm [13], for finding all shortest paths.

The combinatorial complexity of Elementary Modes of a network was previously described by Klamt *et al*. [14] by providing an upper-bound for the number of EMs. In their work, the authors considered the following combinatoric problem: given a network with *n *reactions and *m *metabolites, the maximal number of independent pathways occurs when each possible subset of the reactions consisting of the *m *metabolites are independent. This maximal number was found to be (nm+1)
 MathType@MTEF@5@5@+=feaafiart1ev1aaatCvAUfKttLearuWrP9MDH5MBPbIqV92AaeXatLxBI9gBaebbnrfifHhDYfgasaacH8akY=wiFfYdH8Gipec8Eeeu0xXdbba9frFj0=OqFfea0dXdd9vqai=hGuQ8kuc9pgc9s8qqaq=dirpe0xb9q8qiLsFr0=vr0=vr0dc8meaabaqaciaacaGaaeqabaqabeGadaaakeaadaqadaqaauaabeqaceaaaeaacqWGUbGBaeaacqWGTbqBcqGHRaWkcqaIXaqmaaaacaGLOaGaayzkaaaaaa@32DC@. They further improved the upper-bound by removing those reactions that were not utilized (redundant reactions) in the condition-specific models of the network. Klamt *et al*. generated 5 models of the *E. coli *reconstruction, which yielded 599 to 507632 elementary modes. The upper-bounds for these models ranged from 5.57 × 10^17 ^to 4.39 × 10^21^, which were subsequently reduced to 1.67 × 10^11 ^to 4.85 × 10^13 ^after removing redundant reactions identified using *FluxAnalyzer*. Despite an improvement of a factor of 6 to 8, when comparing the upper-bounds to the actual number of EMs calculated for the models, they typically overestimated by approximately 6 × 10^9^% [14]. Although their work dealt with EMs, the problem was constructed as a purely combinatoric problem. Therefore the same reasoning can be directly applied to Extreme Pathways.

In this study, we investigated the relationship between the number of ExPas for a given network, *p *= |{**p**_*i*_}|, and its basic network measurements. Several network measurements are commonly used in describing the topological features of a network and include connectivity, clustering coefficient, network diameter, and degree distribution [15]. The number of ExPas for a network can vary dramatically under different simulation conditions, that is, different environmental constraints. Consequently, basic network information such as the numbers of reactions and metabolites of the network cannot be solely used to provide a meaningful estimate. Since ExPas are connected reactions, we hypothesized that the higher the reaction connections, the larger the number of ExPas. Based on this hypothesis, we demonstrated an exponential relationship between log [*p*] and log⁡[∑i=1Rd−id+ici]
 MathType@MTEF@5@5@+=feaafiart1ev1aaatCvAUfKttLearuWrP9MDH5MBPbIqV92AaeXatLxBI9gBaebbnrfifHhDYfgasaacH8akY=wiFfYdH8Gipec8Eeeu0xXdbba9frFj0=OqFfea0dXdd9vqai=hGuQ8kuc9pgc9s8qqaq=dirpe0xb9q8qiLsFr0=vr0=vr0dc8meaabaqaciaacaGaaeqabaqabeGadaaakeaacyGGSbaBcqGGVbWBcqGGNbWzdaWadaqaamaaqadabaGaemizaq2aaSbaaSqaaiabgkHiTmaaBaaameaacqWGPbqAaeqaaaWcbeaakiabdsgaKnaaBaaaleaacqGHRaWkdaWgaaadbaGaemyAaKgabeaaaSqabaGccqWGJbWydaWgaaWcbaGaemyAaKgabeaaaeaacqWGPbqAcqGH9aqpcqaIXaqmaeaacqWGsbGua0GaeyyeIuoaaOGaay5waiaaw2faaaaa@4414@, where *R *= |*R*_*eff*_| is the active (or effective) reactions in the network, d−i
 MathType@MTEF@5@5@+=feaafiart1ev1aaatCvAUfKttLearuWrP9MDH5MBPbIqV92AaeXatLxBI9gBaebbnrfifHhDYfgasaacH8akY=wiFfYdH8Gipec8Eeeu0xXdbba9frFj0=OqFfea0dXdd9vqai=hGuQ8kuc9pgc9s8qqaq=dirpe0xb9q8qiLsFr0=vr0=vr0dc8meaabaqaciaacaGaaeqabaqabeGadaaakeaacqWGKbazdaWgaaWcbaGaeyOeI0YaaSbaaWqaaiabdMgaPbqabaaaleqaaaaa@30A9@ and d+i
 MathType@MTEF@5@5@+=feaafiart1ev1aaatCvAUfKttLearuWrP9MDH5MBPbIqV92AaeXatLxBI9gBaebbnrfifHhDYfgasaacH8akY=wiFfYdH8Gipec8Eeeu0xXdbba9frFj0=OqFfea0dXdd9vqai=hGuQ8kuc9pgc9s8qqaq=dirpe0xb9q8qiLsFr0=vr0=vr0dc8meaabaqaciaacaGaaeqabaqabeGadaaakeaacqWGKbazdaWgaaWcbaGaey4kaSYaaSbaaWqaaiabdMgaPbqabaaaleqaaaaa@309E@ are the incoming and outgoing degrees of a reaction, and *c*_*i *_is the clustering coefficient for each active reaction. This relationship typically gave an estimate of the number of ExPas to within a factor of 10. Since these network measurements can be calculated quickly and easily for any sized network, an estimation of ExPa numbers can be readily obtained as this serves as an assessment of the feasibility of ExPas as an analysis tool.

## Results

The number of extreme pathways (ExPa), *p *= |{**p**_*i*_}|, for a metabolic network increases drastically with the complexity and size of the network. An estimate for *p *for a given network can help one decide whether ExPa analysis is a feasible tool for one's research objective. In this study, we investigated the relationship between *p *and a number of factors, *θ*_*i*_, formed by simple network measurements such as the incoming and outgoing degree of reactions, *d*_∓_(*r*_*i*_) = d∓i
 MathType@MTEF@5@5@+=feaafiart1ev1aaatCvAUfKttLearuWrP9MDH5MBPbIqV92AaeXatLxBI9gBaebbnrfifHhDYfgasaacH8akY=wiFfYdH8Gipec8Eeeu0xXdbba9frFj0=OqFfea0dXdd9vqai=hGuQ8kuc9pgc9s8qqaq=dirpe0xb9q8qiLsFr0=vr0=vr0dc8meaabaqaciaacaGaaeqabaqabeGadaaakeaacqWGKbazdaWgaaWcbaGaeS4eI02aaSbaaWqaaiabdMgaPbqabaaaleqaaaaa@30EF@, and clustering coefficient of each reaction, *c*(*r*_*i*_) = *c*_*i*_. These measurements were chosen as they could be calculated quickly and easily, and their definitions can be found in the sections titled 'Reaction Connectivity *d*_±_(*r*_*i*_) = d±i
 MathType@MTEF@5@5@+=feaafiart1ev1aaatCvAUfKttLearuWrP9MDH5MBPbIqV92AaeXatLxBI9gBaebbnrfifHhDYfgasaacH8akY=wiFfYdH8Gipec8Eeeu0xXdbba9frFj0=OqFfea0dXdd9vqai=hGuQ8kuc9pgc9s8qqaq=dirpe0xb9q8qiLsFr0=vr0=vr0dc8meaabaqaciaacaGaaeqabaqabeGadaaakeaacqWGKbazdaWgaaWcbaGaeyySae7aaSbaaWqaaiabdMgaPbqabaaaleqaaaaa@31AA@' and 'Reaction Clustering Coefficient *c*_*i *_= *c*(*r*_*i*_)', respectively. A total of 52 models, generated from 6 reconstructed metabolic networks by altering environmental conditions, were used to determine a relationship between *p *and *θ*_*i*_. The ExPas and corresponding network measurements for these models were calculated and used to identify possible estimating functions. Analyses on logged data (the logarithmic values of the data) revealed an exponential relationship between log [*p*] and log [*θ*_*i*_]. A total of 4 estimating functions using two factors were obtained, which were then tested for robustness using an additional 16 models. The numbers of ExPas for most of these models were successfully estimated to within a factor of 10. We concluded that it was possible to formulate an estimating function for the number of ExPas of a model, *p*', that typically falls within a factor of 10 of the actual number of ExPa.

### Identification of Significant Contributing Factors

We aimed to identify factors that can be used for establishing appropriate estimating functions. Desirable factors must be *i) *easily obtained and *ii) *specific for a given model. For example, network measurements such as the incoming and outgoing degrees of each reaction, *d*_∓_(*r*_*i*_) = d∓i
 MathType@MTEF@5@5@+=feaafiart1ev1aaatCvAUfKttLearuWrP9MDH5MBPbIqV92AaeXatLxBI9gBaebbnrfifHhDYfgasaacH8akY=wiFfYdH8Gipec8Eeeu0xXdbba9frFj0=OqFfea0dXdd9vqai=hGuQ8kuc9pgc9s8qqaq=dirpe0xb9q8qiLsFr0=vr0=vr0dc8meaabaqaciaacaGaaeqabaqabeGadaaakeaacqWGKbazdaWgaaWcbaGaeS4eI02aaSbaaWqaaiabdMgaPbqabaaaleqaaaaa@30EF@, their clustering coefficients, *c*(*r*_*i*_) = *c*_*i*_, as well as the number of external metabolites and their degrees can be obtained quickly. In addition, we required factors to be highly correlated to the number of ExPas and, furthermore, increased in values consistently with *p *to avoid misrepresentation due to incomparable ranges. The network measurements used in this study are detailed in the 'Network Measurements' section.

A number of potential factors for the estimating functions were formed using the aforementioned network measurements (Table 1). The correlations of these potential factors and *p *were evaluated using the Pearson's product-moment correlation, *r*, and Spearman-rank correlation, *ρ*. The Pearson's correlation is generally used as an indicator for the strength and direction of a *linear *relationship and is considered to be robust enough to handle non-parametric data. On the other hand, the Spearman's correlation describes the monotonic relationship between two variables without making any assumptions about the frequency distribution of the variables. We used both of these correlation coefficients on the original and logged data to avoid misinterpretation due to the wide ranges of data (Table 1). For the original data, the factor with the highest Pearson correlation before was found for θ1=∑i=1Rd−id+ici
 MathType@MTEF@5@5@+=feaafiart1ev1aaatCvAUfKttLearuWrP9MDH5MBPbIqV92AaeXatLxBI9gBaebbnrfifHhDYfgasaacH8akY=wiFfYdH8Gipec8Eeeu0xXdbba9frFj0=OqFfea0dXdd9vqai=hGuQ8kuc9pgc9s8qqaq=dirpe0xb9q8qiLsFr0=vr0=vr0dc8meaabaqaciaacaGaaeqabaqabeGadaaakeaaiiGacqWF4oqCdaWgaaWcbaGaeGymaedabeaakiabg2da9maaqadabaGaemizaq2aaSbaaSqaaiabgkHiTmaaBaaameaacqWGPbqAaeqaaaWcbeaakiabdsgaKnaaBaaaleaacqGHRaWkdaWgaaadbaGaemyAaKgabeaaaSqabaGccqWGJbWydaWgaaWcbaGaemyAaKgabeaaaeaacqWGPbqAcqGH9aqpcqaIXaqmaeaacqWGsbGua0GaeyyeIuoaaaa@41E3@, where *R *= |*R*_*eff*_| is the number of active reactions in the network. In contrast, after data-logging, the strongest correlation with *p *was found for θ2=∑i=1Rd−id+i
 MathType@MTEF@5@5@+=feaafiart1ev1aaatCvAUfKttLearuWrP9MDH5MBPbIqV92AaeXatLxBI9gBaebbnrfifHhDYfgasaacH8akY=wiFfYdH8Gipec8Eeeu0xXdbba9frFj0=OqFfea0dXdd9vqai=hGuQ8kuc9pgc9s8qqaq=dirpe0xb9q8qiLsFr0=vr0=vr0dc8meaabaqaciaacaGaaeqabaqabeGadaaakeaaiiGacqWF4oqCdaWgaaWcbaGaeGOmaidabeaakiabg2da9maaqadabaGaemizaq2aaSbaaSqaaiabgkHiTmaaBaaameaacqWGPbqAaeqaaaWcbeaakiabdsgaKnaaBaaaleaacqGHRaWkdaWgaaadbaGaemyAaKgabeaaaSqabaaabaGaemyAaKMaeyypa0JaeGymaedabaGaemOuaifaniabggHiLdaaaa@3F05@. The correlation between these two factors themselves were extremely high (~0.992176). In addition, both factors had the second highest Spearman correlations with *p*. Furthermore, these two factors also had ranges comparable to that of the number of ExPas. Despite the fact that the factors *θ*_1 _and *θ*_2 _are so closely correlated, both of these factors were used to create estimates utilizing single factors in the following section.

**Table 1:** Identification of Potential Contributing Factors

		*R*	* ∑(*d*_+_*d*_-_)	∑*c*	* ∑(*d*_+_*d*_-_*c*)	∑(d+d−)R MathType@MTEF@5@5@+=feaafiart1ev1aaatCvAUfKttLearuWrP9MDH5MBPbIqV92AaeXatLxBI9gBaebbnrfifHhDYfgasaacH8akY=wiFfYdH8Gipec8Eeeu0xXdbba9frFj0=OqFfea0dXdd9vqai=hGuQ8kuc9pgc9s8qqaq=dirpe0xb9q8qiLsFr0=vr0=vr0dc8meaabaqaciaacaGaaeqabaqabeGadaaakeaadaWcaaqaamaaqaeabaGaeiikaGIaemizaq2aaSbaaSqaaiabgUcaRaqabaGccqWGKbazdaWgaaWcbaGaeyOeI0cabeaakiabcMcaPaWcbeqab0GaeyyeIuoaaOqaaiabdkfasbaaaaa@3696@	∑ciR MathType@MTEF@5@5@+=feaafiart1ev1aaatCvAUfKttLearuWrP9MDH5MBPbIqV92AaeXatLxBI9gBaebbnrfifHhDYfgasaacH8akY=wiFfYdH8Gipec8Eeeu0xXdbba9frFj0=OqFfea0dXdd9vqai=hGuQ8kuc9pgc9s8qqaq=dirpe0xb9q8qiLsFr0=vr0=vr0dc8meaabaqaciaacaGaaeqabaqabeGadaaakeaadaWcaaqaamaaqaeabaGaem4yam2aaSbaaSqaaiabdMgaPbqabaaabeqab0GaeyyeIuoaaOqaaiabdkfasbaaaaa@32D2@	∑(d+d−c)R MathType@MTEF@5@5@+=feaafiart1ev1aaatCvAUfKttLearuWrP9MDH5MBPbIqV92AaeXatLxBI9gBaebbnrfifHhDYfgasaacH8akY=wiFfYdH8Gipec8Eeeu0xXdbba9frFj0=OqFfea0dXdd9vqai=hGuQ8kuc9pgc9s8qqaq=dirpe0xb9q8qiLsFr0=vr0=vr0dc8meaabaqaciaacaGaaeqabaqabeGadaaakeaadaWcaaqaamaaqaeabaGaeiikaGIaemizaq2aaSbaaSqaaiabgUcaRaqabaGccqWGKbazdaWgaaWcbaGaeyOeI0cabeaakiabdogaJjabcMcaPaWcbeqab0GaeyyeIuoaaOqaaiabdkfasbaaaaa@37E5@	∑ℐdi MathType@MTEF@5@5@+=feaafiart1ev1aaatCvAUfKttLearuWrP9MDH5MBPbIqV92AaeXatLxBI9gBaebbnrfifHhDYfgasaacH8akY=wiFfYdH8Gipec8Eeeu0xXdbba9frFj0=OqFfea0dXdd9vqai=hGuQ8kuc9pgc9s8qqaq=dirpe0xb9q8qiLsFr0=vr0=vr0dc8meaabaqaciaacaGaaeqabaqabeGadaaakeaadaaeqaqaaiabdsgaKnaaBaaaleaacqWGPbqAaeqaaaqaamrtHrhAL1wy0L2yHvtyaeHbnfgDOvwBHrxAJfwnaGabaiab=brijbqab0GaeyyeIuoaaaa@3C18@	∑Od MathType@MTEF@5@5@+=feaafiart1ev1aaatCvAUfKttLearuWrP9MDH5MBPbIqV92AaeXatLxBI9gBaebbnrfifHhDYfgasaacH8akY=wiFfYdH8Gipec8Eeeu0xXdbba9frFj0=OqFfea0dXdd9vqai=hGuQ8kuc9pgc9s8qqaq=dirpe0xb9q8qiLsFr0=vr0=vr0dc8meaabaqaciaacaGaaeqabaqabeGadaaakeaadaaeqaqaaiabdsgaKbWcbaWenfgDOvwBHrxAJfwnHbqeg0uy0HwzTfgDPnwy1aaceaGae8NdX=eabeqdcqGHris5aaaa@3B70@	(∑ℐd)(∑Od) MathType@MTEF@5@5@+=feaafiart1ev1aaatCvAUfKttLearuWrP9MDH5MBPbIqV92AaeXatLxBI9gBaebbnrfifHhDYfgasaacH8akY=wiFfYdH8Gipec8Eeeu0xXdbba9frFj0=OqFfea0dXdd9vqai=hGuQ8kuc9pgc9s8qqaq=dirpe0xb9q8qiLsFr0=vr0=vr0dc8meaabaqaciaacaGaaeqabaqabeGadaaakeaadaqadaqaamaaqababaGaemizaqgaleaat0uy0HwzTfgDPnwy1egaryqtHrhAL1wy0L2yHvdaiqaacqWFqessaeqaniabggHiLdaakiaawIcacaGLPaaadaqadaqaamaaqababaGaemizaqgaleaacqWFoe=taeqaniabggHiLdaakiaawIcacaGLPaaaaaa@42C9@
***r *Pre-log**		0.459	0.656	0.509	0.666	0.6	0.0178	0.62	0.135	0.604	0.408
***r *Post-log**		0.827	0.875	0.764	0.870	0.860	0.343	0.856	0.426	0.561	0.623
***ρ***		0.841	0.876	0.943	0.876	0.855	-0.059	0.845	0.478	0.496	0.603
**Range**	**Min**	8	15	5	2	1.67	0.09	0.06	2	2	6
	**Max**	174	89132	35.08	5781.73	665.38	0.9	47.78	25	47	414

A good factor for an estimating function must have a high correlation to that is being estimated. We further required that the factor must grow consistently with the number of ExPas. The rows labelled 'Pre-' and 'Post-' Log show the Pearson's correlations, *r*, between the number of ExPas and the corresponding factors. These factors were created using the following basic networks measurements: *R *= *R*_*eff *_the number of active reactions given the environmental conditions, *d*_± _= *d*_±_(*r*_*i*_) the incoming/outgoing connectivity of reaction *r*_*i*_, *c *= *c*(*r*_*i*_) the clustering coefficient of the *i*^*th *^reaction, ℐ
 MathType@MTEF@5@5@+=feaafiart1ev1aaatCvAUfKttLearuWrP9MDH5MBPbIqV92AaeXatLxBI9gBaebbnrfifHhDYfgasaacH8akY=wiFfYdH8Gipec8Eeeu0xXdbba9frFj0=OqFfea0dXdd9vqai=hGuQ8kuc9pgc9s8qqaq=dirpe0xb9q8qiLsFr0=vr0=vr0dc8meaabaqaciaacaGaaeqabaqabeGadaaakeaat0uy0HwzTfgDPnwy1egaryqtHrhAL1wy0L2yHvdaiqaaliab=brijbaa@3773@ the set of input reactions, and O
 MathType@MTEF@5@5@+=feaafiart1ev1aaatCvAUfKttLearuWrP9MDH5MBPbIqV92AaeXatLxBI9gBaebbnrfifHhDYfgasaacH8akY=wiFfYdH8Gipec8Eeeu0xXdbba9frFj0=OqFfea0dXdd9vqai=hGuQ8kuc9pgc9s8qqaq=dirpe0xb9q8qiLsFr0=vr0=vr0dc8meaabaqaciaacaGaaeqabaqabeGadaaakeaat0uy0HwzTfgDPnwy1egaryqtHrhAL1wy0L2yHvdaiqaaliab=5q8pbaa@3847@ the set of output reactions. Both Pearson's and Spearman's Rank correlation coefficients, *r *and *ρ *were used a guide to identify reliable contributing factors. Given this information, the final chosen factors are emphasized by an asterisk (*).

### Single Factor Estimate

**Figure 1:**
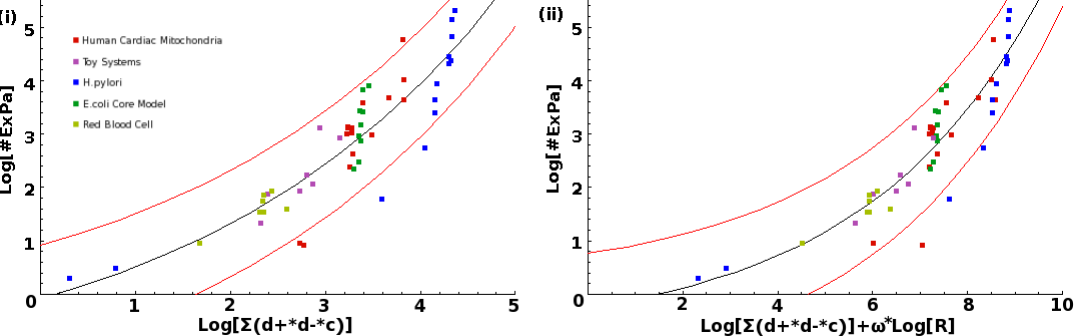
**Relationship between the Number of ExPas and Factor **θ1=∑i=1Rd−id+ici
 MathType@MTEF@5@5@+=feaafiart1ev1aaatCvAUfKttLearuWrP9MDH5MBPbIqV92AaeXatLxBI9gBaebbnrfifHhDYfgasaacH8akY=wiFfYdH8Gipec8Eeeu0xXdbba9frFj0=OqFfea0dXdd9vqai=hGuQ8kuc9pgc9s8qqaq=dirpe0xb9q8qiLsFr0=vr0=vr0dc8meaabaqaciaacaGaaeqabaqabeGadaaakeaaiiGacqWF4oqCdaWgaaWcbaGaeGymaedabeaakiabg2da9maaqadabaGaemizaq2aaSbaaSqaaiabgkHiTmaaBaaameaacqWGPbqAaeqaaaWcbeaakiabdsgaKnaaBaaaleaacqGHRaWkdaWgaaadbaGaemyAaKgabeaaaSqabaGccqWGJbWydaWgaaWcbaGaemyAaKgabeaaaeaacqWGPbqAcqGH9aqpcqaIXaqmaeaacqWGsbGua0GaeyyeIuoaaaa@41E3@. Graphs displaying the two relationships derived from the factor θ1=∑i=1Rd−id+ici
 MathType@MTEF@5@5@+=feaafiart1ev1aaatCvAUfKttLearuWrP9MDH5MBPbIqV92AaeXatLxBI9gBaebbnrfifHhDYfgasaacH8akY=wiFfYdH8Gipec8Eeeu0xXdbba9frFj0=OqFfea0dXdd9vqai=hGuQ8kuc9pgc9s8qqaq=dirpe0xb9q8qiLsFr0=vr0=vr0dc8meaabaqaciaacaGaaeqabaqabeGadaaakeaaiiGacqWF4oqCdaWgaaWcbaGaeGymaedabeaakiabg2da9maaqadabaGaemizaq2aaSbaaSqaaiabgkHiTmaaBaaameaacqWGPbqAaeqaaaWcbeaakiabdsgaKnaaBaaaleaacqGHRaWkdaWgaaadbaGaemyAaKgabeaaaSqabaGccqWGJbWydaWgaaWcbaGaemyAaKgabeaaaeaacqWGPbqAcqGH9aqpcqaIXaqmaeaacqWGsbGua0GaeyyeIuoaaaa@41E3@. It was observed that *θ*_1 _had an exponential relation to *p *as shown in (i). The use of *R*^*ω *^as a scaling factor was found to improve the fit of the data (ii).

**Figure 2:**
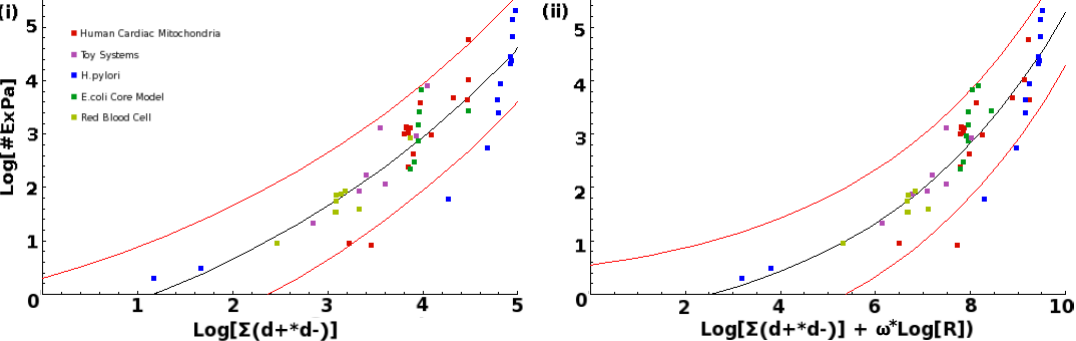
**Relationship between the Number of ExPas and Factor **θ2=∑i=1Rd−id+i
 MathType@MTEF@5@5@+=feaafiart1ev1aaatCvAUfKttLearuWrP9MDH5MBPbIqV92AaeXatLxBI9gBaebbnrfifHhDYfgasaacH8akY=wiFfYdH8Gipec8Eeeu0xXdbba9frFj0=OqFfea0dXdd9vqai=hGuQ8kuc9pgc9s8qqaq=dirpe0xb9q8qiLsFr0=vr0=vr0dc8meaabaqaciaacaGaaeqabaqabeGadaaakeaaiiGacqWF4oqCdaWgaaWcbaGaeGOmaidabeaakiabg2da9maaqadabaGaemizaq2aaSbaaSqaaiabgkHiTmaaBaaameaacqWGPbqAaeqaaaWcbeaakiabdsgaKnaaBaaaleaacqGHRaWkdaWgaaadbaGaemyAaKgabeaaaSqabaaabaGaemyAaKMaeyypa0JaeGymaedabaGaemOuaifaniabggHiLdaaaa@3F05@. Similar to Figure 1, it was observed that θ2=∑i=1Rd−id+i
 MathType@MTEF@5@5@+=feaafiart1ev1aaatCvAUfKttLearuWrP9MDH5MBPbIqV92AaeXatLxBI9gBaebbnrfifHhDYfgasaacH8akY=wiFfYdH8Gipec8Eeeu0xXdbba9frFj0=OqFfea0dXdd9vqai=hGuQ8kuc9pgc9s8qqaq=dirpe0xb9q8qiLsFr0=vr0=vr0dc8meaabaqaciaacaGaaeqabaqabeGadaaakeaaiiGacqWF4oqCdaWgaaWcbaGaeGOmaidabeaakiabg2da9maaqadabaGaemizaq2aaSbaaSqaaiabgkHiTmaaBaaameaacqWGPbqAaeqaaaWcbeaakiabdsgaKnaaBaaaleaacqGHRaWkdaWgaaadbaGaemyAaKgabeaaaSqabaaabaGaemyAaKMaeyypa0JaeGymaedabaGaemOuaifaniabggHiLdaaaa@3F05@ also had an exponential relation to log [*p*] (i), which could be improved if scaled by *R*^*ω *^(ii).

The factors identified in the previous section, namely θ1=∑i=1Rd−id+ici
 MathType@MTEF@5@5@+=feaafiart1ev1aaatCvAUfKttLearuWrP9MDH5MBPbIqV92AaeXatLxBI9gBaebbnrfifHhDYfgasaacH8akY=wiFfYdH8Gipec8Eeeu0xXdbba9frFj0=OqFfea0dXdd9vqai=hGuQ8kuc9pgc9s8qqaq=dirpe0xb9q8qiLsFr0=vr0=vr0dc8meaabaqaciaacaGaaeqabaqabeGadaaakeaaiiGacqWF4oqCdaWgaaWcbaGaeGymaedabeaakiabg2da9maaqadabaGaemizaq2aaSbaaSqaaiabgkHiTmaaBaaameaacqWGPbqAaeqaaaWcbeaakiabdsgaKnaaBaaaleaacqGHRaWkdaWgaaadbaGaemyAaKgabeaaaSqabaGccqWGJbWydaWgaaWcbaGaemyAaKgabeaaaeaacqWGPbqAcqGH9aqpcqaIXaqmaeaacqWGsbGua0GaeyyeIuoaaaa@41E3@ and θ2=∑i=1Rd−id+i
 MathType@MTEF@5@5@+=feaafiart1ev1aaatCvAUfKttLearuWrP9MDH5MBPbIqV92AaeXatLxBI9gBaebbnrfifHhDYfgasaacH8akY=wiFfYdH8Gipec8Eeeu0xXdbba9frFj0=OqFfea0dXdd9vqai=hGuQ8kuc9pgc9s8qqaq=dirpe0xb9q8qiLsFr0=vr0=vr0dc8meaabaqaciaacaGaaeqabaqabeGadaaakeaaiiGacqWF4oqCdaWgaaWcbaGaeGOmaidabeaakiabg2da9maaqadabaGaemizaq2aaSbaaSqaaiabgkHiTmaaBaaameaacqWGPbqAaeqaaaWcbeaakiabdsgaKnaaBaaaleaacqGHRaWkdaWgaaadbaGaemyAaKgabeaaaSqabaaabaGaemyAaKMaeyypa0JaeGymaedabaGaemOuaifaniabggHiLdaaaa@3F05@ were used to formulate estimating functions for the number of extreme pathways, *p*. Preliminary analyses showed that the relationships between log [*p*] and log [*θ*_*j*_], *j *= 1, 2, were non-linear (Figure 1 and Figure 2). In particular, the expression found to best describe these relationships had the form

log⁡[p^k]=fi(θj,k)=αi,j+βi,j10γi,j(ωilog⁡[R]+log⁡[θj,k])=αi,j+βi,j(Rkωiθj,k)γi,j,i=1,2,
 MathType@MTEF@5@5@+=feaafiart1ev1aaatCvAUfKttLearuWrP9MDH5MBPbIqV92AaeXatLxBI9gBaebbnrfifHhDYfgasaacH8akY=wiFfYdH8Gipec8Eeeu0xXdbba9frFj0=OqFfea0dXdd9vqai=hGuQ8kuc9pgc9s8qqaq=dirpe0xb9q8qiLsFr0=vr0=vr0dc8meaabaqaciaacaGaaeqabaqabeGadaaakeaafaqabeGadaaabaGagiiBaWMaei4Ba8Maei4zaCMaei4waSLafmiCaaNbaKaadaWgaaWcbaGaem4AaSgabeaakiabc2faDjabg2da9iabdAgaMnaaBaaaleaacqWGPbqAaeqaaOGaeiikaGccciGae8hUde3aaSbaaSqaaiabdQgaQjabcYcaSiabdUgaRbqabaGccqGGPaqkaeaacqGH9aqpaeaacqWFXoqydaWgaaWcbaGaemyAaKMaeiilaWIaemOAaOgabeaakiabgUcaRiab=j7aInaaBaaaleaacqWGPbqAcqGGSaalcqWGQbGAaeqaaOGaeGymaeJaeGimaaZaaWbaaSqabeaacqWFZoWzdaWgaaadbaGaemyAaKMaeiilaWIaemOAaOgabeaaliabcIcaOiab=L8a3naaBaaameaacqWGPbqAaeqaaSGagiiBaWMaei4Ba8Maei4zaCMaei4waSLaemOuaiLaeiyxa0Laey4kaSIagiiBaWMaei4Ba8Maei4zaCMaei4waSLae8hUde3aaSbaaWqaaiabdQgaQjabcYcaSiabdUgaRbqabaWccqGGDbqxcqGGPaqkaaaakeaaaeaacqGH9aqpaeaafaqabeqacaaabaGae8xSde2aaSbaaSqaaiabdMgaPjabcYcaSiabdQgaQbqabaGccqGHRaWkcqWFYoGydaWgaaWcbaGaemyAaKMaeiilaWIaemOAaOgabeaakmaabmaabaGaemOuai1aa0baaSqaaiabdUgaRbqaaiab=L8a3naaBaaameaacqWGPbqAaeqaaaaakiab=H7aXnaaBaaaleaacqWGQbGAcqGGSaalcqWGRbWAaeqaaaGccaGLOaGaayzkaaWaaWbaaSqabeaacqWFZoWzdaWgaaadbaGaemyAaKMaeiilaWIaemOAaOgabeaaaaGccqGGSaalaeaacqWGPbqAcqGH9aqpcqaIXaqmcqGGSaalcqaIYaGmcqGGSaalaaaaaaaa@961D@

where p^k
 MathType@MTEF@5@5@+=feaafiart1ev1aaatCvAUfKttLearuWrP9MDH5MBPbIqV92AaeXatLxBI9gBaebbnrfifHhDYfgasaacH8akY=wiFfYdH8Gipec8Eeeu0xXdbba9frFj0=OqFfea0dXdd9vqai=hGuQ8kuc9pgc9s8qqaq=dirpe0xb9q8qiLsFr0=vr0=vr0dc8meaabaqaciaacaGaaeqabaqabeGadaaakeaacuWGWbaCgaqcamaaBaaaleaacqWGRbWAaeqaaaaa@2FB0@ is the estimated number of ExPas for a given model *k*, *θ*_*j*, *k *_are the values of the factors *θ*_*j*_, *j *= 1, 2 for the *k*^th ^model, and *R*_*k *_is the number of active reactions in model *k*. The estimating functions were formulated using the factors *θ*_*j *_solely (case *i *= 1) and by scaling these factors with Rkω
 MathType@MTEF@5@5@+=feaafiart1ev1aaatCvAUfKttLearuWrP9MDH5MBPbIqV92AaeXatLxBI9gBaebbnrfifHhDYfgasaacH8akY=wiFfYdH8Gipec8Eeeu0xXdbba9frFj0=OqFfea0dXdd9vqai=hGuQ8kuc9pgc9s8qqaq=dirpe0xb9q8qiLsFr0=vr0=vr0dc8meaabaqaciaacaGaaeqabaqabeGadaaakeaacqWGsbGudaqhaaWcbaGaem4AaSgabaacciGae8xYdChaaaaa@3139@ (case *i *= 2). For the former, the parameter *ω*_1 _had the value 0. For the latter case, *ω*_2 _was the optimal value for which the highest Pearson's correlation between log [*p*] and log [p^k
 MathType@MTEF@5@5@+=feaafiart1ev1aaatCvAUfKttLearuWrP9MDH5MBPbIqV92AaeXatLxBI9gBaebbnrfifHhDYfgasaacH8akY=wiFfYdH8Gipec8Eeeu0xXdbba9frFj0=OqFfea0dXdd9vqai=hGuQ8kuc9pgc9s8qqaq=dirpe0xb9q8qiLsFr0=vr0=vr0dc8meaabaqaciaacaGaaeqabaqabeGadaaakeaacuWGWbaCgaqcamaaBaaaleaacqWGRbWAaeqaaaaa@2FB0@] could be obtained using the two different factors. This number was found to be the same for both factors and had the value *ω*_2 _= 2.124857. The parameters *α*_*i*, *j*_, *β*_*i*, *j*_, and *γ*_*i*, *j *_take on different values for the four estimating functions *f*_*i *_(*θ*_*j*_), which are detailed in the following subsections 'Estimation Using *θ*_1_' and 'Estimation Using *θ*_2_'.

#### Estimation Using *θ*_1_

Using factor θ1=∑i=1Rd−id+ici
 MathType@MTEF@5@5@+=feaafiart1ev1aaatCvAUfKttLearuWrP9MDH5MBPbIqV92AaeXatLxBI9gBaebbnrfifHhDYfgasaacH8akY=wiFfYdH8Gipec8Eeeu0xXdbba9frFj0=OqFfea0dXdd9vqai=hGuQ8kuc9pgc9s8qqaq=dirpe0xb9q8qiLsFr0=vr0=vr0dc8meaabaqaciaacaGaaeqabaqabeGadaaakeaaiiGacqWF4oqCdaWgaaWcbaGaeGymaedabeaakiabg2da9maaqadabaGaemizaq2aaSbaaSqaaiabgkHiTmaaBaaameaacqWGPbqAaeqaaaWcbeaakiabdsgaKnaaBaaaleaacqGHRaWkdaWgaaadbaGaemyAaKgabeaaaSqabaGccqWGJbWydaWgaaWcbaGaemyAaKgabeaaaeaacqWGPbqAcqGH9aqpcqaIXaqmaeaacqWGsbGua0GaeyyeIuoaaaa@41E3@, the following estimating function was obtained when *ω*_1 _was applied:

f1(θ1,k)=−1.708123+1.624207(∑i=1Rkd−id+ici)0.135324,
 MathType@MTEF@5@5@+=feaafiart1ev1aaatCvAUfKttLearuWrP9MDH5MBPbIqV92AaeXatLxBI9gBaebbnrfifHhDYfgasaacH8akY=wiFfYdH8Gipec8Eeeu0xXdbba9frFj0=OqFfea0dXdd9vqai=hGuQ8kuc9pgc9s8qqaq=dirpe0xb9q8qiLsFr0=vr0=vr0dc8meaabaqaciaacaGaaeqabaqabeGadaaakeaacqWGMbGzdaWgaaWcbaGaeGymaedabeaakiabcIcaOGGaciab=H7aXnaaBaaaleaacqaIXaqmcqGGSaalcqWGRbWAaeqaaOGaeiykaKIaeyypa0JaeyOeI0IaeGymaeJaeiOla4IaeG4naCJaeGimaaJaeGioaGJaeGymaeJaeGOmaiJaeG4mamJaey4kaSIaeGymaeJaeiOla4IaeGOnayJaeGOmaiJaeGinaqJaeGOmaiJaeGimaaJaeG4naCZaaeWaaeaadaaeWbqaaiabdsgaKnaaBaaaleaacqGHsisldaWgaaadbaGaemyAaKgabeaaaSqabaGccqWGKbazdaWgaaWcbaGaey4kaSYaaSbaaWqaaiabdMgaPbqabaaaleqaaOGaem4yam2aaSbaaSqaaiabdMgaPbqabaaabaGaemyAaKMaeyypa0JaeGymaedabaGaemOuai1aaSbaaWqaaiabdUgaRbqabaaaniabggHiLdaakiaawIcacaGLPaaadaahaaWcbeqaaiabicdaWiabc6caUiabigdaXiabiodaZiabiwda1iabiodaZiabikdaYiabisda0aaakiabcYcaSaaa@6542@

and with *ω*_2_:

f2(θ1,k)=−0.750588+0.514844(Rk2.124857∑i=1Rd−id+ici)0.114214.
 MathType@MTEF@5@5@+=feaafiart1ev1aaatCvAUfKttLearuWrP9MDH5MBPbIqV92AaeXatLxBI9gBaebbnrfifHhDYfgasaacH8akY=wiFfYdH8Gipec8Eeeu0xXdbba9frFj0=OqFfea0dXdd9vqai=hGuQ8kuc9pgc9s8qqaq=dirpe0xb9q8qiLsFr0=vr0=vr0dc8meaabaqaciaacaGaaeqabaqabeGadaaakeaacqWGMbGzdaWgaaWcbaGaeGOmaidabeaakiabcIcaOGGaciab=H7aXnaaBaaaleaacqaIXaqmcqGGSaalcqWGRbWAaeqaaOGaeiykaKIaeyypa0JaeyOeI0IaeGimaaJaeiOla4IaeG4naCJaeGynauJaeGimaaJaeGynauJaeGioaGJaeGioaGJaey4kaSIaeGimaaJaeiOla4IaeGynauJaeGymaeJaeGinaqJaeGioaGJaeGinaqJaeGinaqZaaeWaaeaacqWGsbGudaqhaaWcbaGaem4AaSgabaGaeGOmaiJaeiOla4IaeGymaeJaeGOmaiJaeGinaqJaeGioaGJaeGynauJaeG4naCdaaOWaaabCaeaacqWGKbazdaWgaaWcbaGaeyOeI0YaaSbaaWqaaiabdMgaPbqabaaaleqaaOGaemizaq2aaSbaaSqaaiabgUcaRmaaBaaameaacqWGPbqAaeqaaaWcbeaakiabdogaJnaaBaaaleaacqWGPbqAaeqaaaqaaiabdMgaPjabg2da9iabigdaXaqaaiabdkfasbqdcqGHris5aaGccaGLOaGaayzkaaWaaWbaaSqabeaacqaIWaamcqGGUaGlcqaIXaqmcqaIXaqmcqaI0aancqaIYaGmcqaIXaqmcqaI0aanaaGccqGGUaGlaaa@6E33@

The fitted curves given by Equations (2) and (3) are shown in Figure 1. The Pearson's correlation was 0.883439 for the function given by Equation (2), whereas that given by Equation(3) resulted in a better fit, with correlation being 0.900704 and reduced mean absolute- and root-mean-square errors (Table 2). The overall performance of this estimator was evaluated. It was found that the number of ExPas for most of the models (47 out of 52) could be described to within a factor of 10 using the estimating functions, while those that could not tended to be over-estimated (Table 2). The inclusion of the factor Rω2
 MathType@MTEF@5@5@+=feaafiart1ev1aaatCvAUfKttLearuWrP9MDH5MBPbIqV92AaeXatLxBI9gBaebbnrfifHhDYfgasaacH8akY=wiFfYdH8Gipec8Eeeu0xXdbba9frFj0=OqFfea0dXdd9vqai=hGuQ8kuc9pgc9s8qqaq=dirpe0xb9q8qiLsFr0=vr0=vr0dc8meaabaqaciaacaGaaeqabaqabeGadaaakeaacqWGsbGudaahaaWcbeqaaGGaciab=L8a3naaBaaameaacqaIYaGmaeqaaaaaaaa@30F9@ led to better fits between the estimating function and the training data and reduced average errors.

**Table 2:** Fit of Training Data to Estimating Functions

	Fit of Factors			Over-Estimation		Under-Estimation	
Factor	Correlation *r*	m.a.e.	r.m.s.	Max. Value	Members>1	Min. Value	Members<1

*θ*_1 _= ∑*d*_+_*d*_-_*c*	0.883439	0.407565	0.539834	1.512650	5	-1.138180	1
*θ*_1 _= ∑*d*_+_*d*_-_	^†^0.887057	0.406882	0.544633	1.585230	3	-1.071100	1
*R*^*ω*^*θ*_1_	^‡^0.900704	0.383868	0.512515	1.629060	3	-0.918259	0
*R*^*ω*^*θ*_1_	0.898332	0.381380	0.518272	1.674300	3	-0.963172	0

This table summarizes the statistics describing the relationships between the four different estimations *f*_*i*_(*θ*_*j*_) and the 52 data points. The results show that the two factors *θ*_1 _and *θ*_2 _allowed better estimations when the scaling factor *R*^*ω *^was included. The top two rows and the bottom two rows of the table, respectively, represent the factors before and after scaling by the factor *R*^*ω*^. The cells with the highest Pearson's correlation, *r*, before and after scaling are emphasized with † and ‡, respectively.

#### Estimation Using *θ*_2_

Using the second factor, *θ*_2_, the estimating functions with and without scaling had the respective forms

f1(θ2)=−2.671743+1.927198(∑i=1Rd−id+i)0.113432
 MathType@MTEF@5@5@+=feaafiart1ev1aaatCvAUfKttLearuWrP9MDH5MBPbIqV92AaeXatLxBI9gBaebbnrfifHhDYfgasaacH8akY=wiFfYdH8Gipec8Eeeu0xXdbba9frFj0=OqFfea0dXdd9vqai=hGuQ8kuc9pgc9s8qqaq=dirpe0xb9q8qiLsFr0=vr0=vr0dc8meaabaqaciaacaGaaeqabaqabeGadaaakeaacqWGMbGzdaWgaaWcbaGaeGymaedabeaakiabcIcaOGGaciab=H7aXnaaBaaaleaacqaIYaGmaeqaaOGaeiykaKIaeyypa0JaeyOeI0IaeGOmaiJaeiOla4IaeGOnayJaeG4naCJaeGymaeJaeG4naCJaeGinaqJaeG4mamJaey4kaSIaeGymaeJaeiOla4IaeGyoaKJaeGOmaiJaeG4naCJaeGymaeJaeGyoaKJaeGioaGZaaeWaaeaadaaeWbqaaiabdsgaKnaaBaaaleaacqGHsisldaWgaaadbaGaemyAaKgabeaaaSqabaGccqWGKbazdaWgaaWcbaGaey4kaSYaaSbaaWqaaiabdMgaPbqabaaaleqaaaqaaiabdMgaPjabg2da9iabigdaXaqaaiabdkfasbqdcqGHris5aaGccaGLOaGaayzkaaWaaWbaaSqabeaacqaIWaamcqGGUaGlcqaIXaqmcqaIXaqmcqaIZaWmcqaI0aancqaIZaWmcqaIYaGmaaaaaa@5DD5@

and

f2(θ2)=−0.971205+0.515711(R2.124857∑i=1Rd−id+i)0.108329.
 MathType@MTEF@5@5@+=feaafiart1ev1aaatCvAUfKttLearuWrP9MDH5MBPbIqV92AaeXatLxBI9gBaebbnrfifHhDYfgasaacH8akY=wiFfYdH8Gipec8Eeeu0xXdbba9frFj0=OqFfea0dXdd9vqai=hGuQ8kuc9pgc9s8qqaq=dirpe0xb9q8qiLsFr0=vr0=vr0dc8meaabaqaciaacaGaaeqabaqabeGadaaakeaacqWGMbGzdaWgaaWcbaGaeGOmaidabeaakiabcIcaOGGaciab=H7aXnaaBaaaleaacqaIYaGmaeqaaOGaeiykaKIaeyypa0JaeyOeI0IaeGimaaJaeiOla4IaeGyoaKJaeG4naCJaeGymaeJaeGOmaiJaeGimaaJaeGynauJaey4kaSIaeGimaaJaeiOla4IaeGynauJaeGymaeJaeGynauJaeG4naCJaeGymaeJaeGymaeZaaeWaaeaacqWGsbGudaahaaWcbeqaaiabikdaYiabc6caUiabigdaXiabikdaYiabisda0iabiIda4iabiwda1iabiEda3aaakmaaqahabaGaemizaq2aaSbaaSqaaiabgkHiTmaaBaaameaacqWGPbqAaeqaaaWcbeaakiabdsgaKnaaBaaaleaacqGHRaWkdaWgaaadbaGaemyAaKgabeaaaSqabaaabaGaemyAaKMaeyypa0JaeGymaedabaGaemOuaifaniabggHiLdaakiaawIcacaGLPaaadaahaaWcbeqaaiabicdaWiabc6caUiabigdaXiabicdaWiabiIda4iabiodaZiabikdaYiabiMda5aaakiabc6caUaaa@67AD@

The relationships between log [*p*] and Equations (4) and (5) are displayed in Figure 2. In this case, the Pearson's correlation before the inclusion of *R*^*ω *^was 0.887057, and was improved to 0.898332 after scaling.

Similar to the case for *θ*_1_, the errors were reduced after scaling (Table 2). The unscaled estimating function, Equation (4), again, described most of the models (48 out of 52) to within a a factor of 10 with respect to the actual ExPa numbers (Table 2). The inclusion of the scaling factor resulted in less outliers, a better correlation and reduced errors (Table 2).

### Performance of Estimations Functions

**Figure 3:**
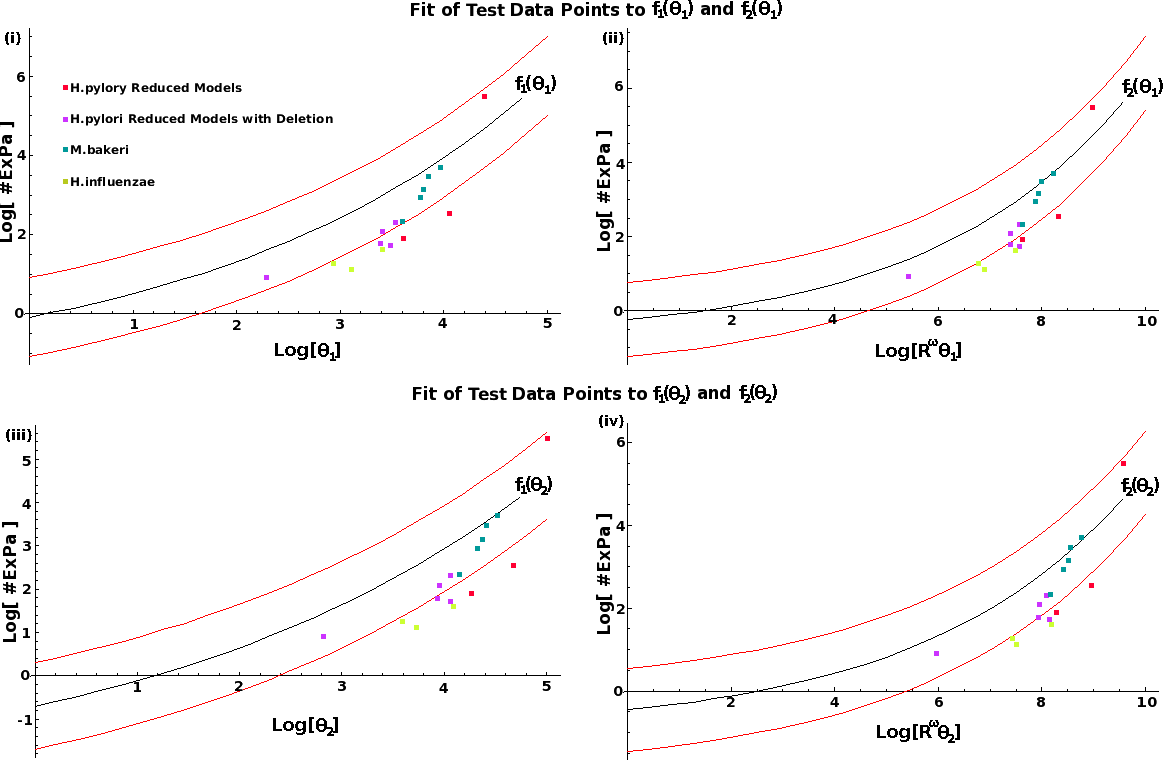
**Comparison of Test Models to the Estimating Functions**. Figures displaying the relationships amongst the test data points and the four estimation functions given by equations (2), (3), (4) and (5). These are shown in (i), (ii), (iii) and (iv) respectively. The red lines in each case are given by *f*_*i*_(*θ*_*j*_) ± 1 and indicate the boundaries of the regions that are within a factor of 10 of the respective estimations.

The performance of the estimating functions (2–5) was tested using an additional 16 models. These models were reduced but functional models of the central metabolism derived from reconstructed metabolic networks of 3 different organisms, namely *H. pylori *[3], *M. barkeri *[16], and *H. influenzae *[1]. All four estimating functions successfully predicted 9 out of the 16 test models (56%) to within a factor of 10 (Figure 3). For all four estimating functions, the number of ExPas of seven models were overestimated by a factor greater than 10 while none were under-estimated beyond that factor. In particular, the estimating function *f*_2_(*θ*_1_) yielded the smallest error range for all models. The 16 test data points had the highest correlation with *f*_2_(*θ*_1_), as did the 52 data points used for its formulation (Table 3). We concluded that the estimating function *f*_2_(*θ*_1_) can typically successfully estimate the number of ExPas of a metabolic network to within a factor of 10.

**Table 3:** Fit of Test Data to Estimating Functions

	Fit of Factors			Over-Estimation		Under-Estimation	
Factor	Correlation *r*	m.a.e.	r.m.s.	Max. Value	Members>1	Min. Value	Members<1

*f*_1 _(*θ*_1_)	0.893058	0.956777	1.042668	1.513670	7	-0.785898	0
*f*_2 _(*θ*_1_)	*0.894445	0.806018	0.919502	1.353102	7	-0.765522	0
*f*_1 _(*θ*_2_)	0.864965	0.891715	1.004652	1.501480	7	-0.851316	0
*f*_2 _(*θ*_2_)	0.876409	0.777881	0.897856	1.401680	7	-0.808617	0

Table detailing the relationships between the 16 test data points and the four estimating functions. Although all four functions failed to predict 7 models to within a factor of 10 of their actual number of ExPas, the function *f*_2 _(*θ*_1_) had the least range for over- and under-estimation. Rows representing information of estimating functions using factors *θ*_1 _and *θ*_2_, respectively, in the top two and bottom two rows. The cell corresponding to the highest correlation, *r*, is emphasized by an asterisk (*).

### Consideration of Other Network Measurements

During the development of these estimating functions, other factors such as the degrees of exchange metabolites were considered. In the case of exchange metabolites, the correlation of the sum of the degrees of all input metabolites, Din=∑i=1|M−|d(mi−)
 MathType@MTEF@5@5@+=feaafiart1ev1aaatCvAUfKttLearuWrP9MDH5MBPbIqV92AaeXatLxBI9gBaebbnrfifHhDYfgasaacH8akY=wiFfYdH8Gipec8Eeeu0xXdbba9frFj0=OqFfea0dXdd9vqai=hGuQ8kuc9pgc9s8qqaq=dirpe0xb9q8qiLsFr0=vr0=vr0dc8meaabaqaciaacaGaaeqabaqabeGadaaakeaacqWGebardaWgaaWcbaGaemyAaKMaemOBa4gabeaakiabg2da9maaqadabaGaemizaqMaeiikaGIaemyBa02aa0baaSqaaiabdMgaPbqaaiabgkHiTaaakiabcMcaPaWcbaGaemyAaKMaeyypa0JaeGymaedabaGaeiiFaWNaemyta00aaWbaaWqabeaacqGHsislaaWccqGG8baFa0GaeyyeIuoaaaa@433A@ was found to have a low correlation (< 0.5) to the number of extreme pathways, both before and after the data was logged. The correlation for output metabolites, Dout=∑i=1|M+|d(mi+)
 MathType@MTEF@5@5@+=feaafiart1ev1aaatCvAUfKttLearuWrP9MDH5MBPbIqV92AaeXatLxBI9gBaebbnrfifHhDYfgasaacH8akY=wiFfYdH8Gipec8Eeeu0xXdbba9frFj0=OqFfea0dXdd9vqai=hGuQ8kuc9pgc9s8qqaq=dirpe0xb9q8qiLsFr0=vr0=vr0dc8meaabaqaciaacaGaaeqabaqabeGadaaakeaacqWGebardaWgaaWcbaGaem4Ba8MaemyDauNaemiDaqhabeaakiabg2da9maaqadabaGaemizaqMaeiikaGIaemyBa02aa0baaSqaaiabdMgaPbqaaiabgUcaRaaakiabcMcaPaWcbaGaemyAaKMaeyypa0JaeGymaedabaGaeiiFaWNaemyta00aaWbaaWqabeaacqGHRaWkaaWccqGG8baFa0GaeyyeIuoaaaa@44AF@, however, was found to have a high correlation (> 0.5) to *p*. Their product, *D*_*in*_·*D*_*out*_, had correlations of 0.408543 and 0.638261 before and after the data was logged. A similar situation existed for the number of effective reactions *R*, which had a low and high correlation before and after the data was logged. By inspection, a relationship in the bform of Equation (1) could have been established for *R*. However, the range of *R *did not grow as consistently as *θ*_1 _and *θ*_2_. As an example, for one *H. pylori *model, 174 effective reactions resulted in 204,412 extreme pathways, whereas for the Mitochondria model with the reaction SUCD3-u10 m removed, 174 effective reactions only produced 4209 ExPas. This then led to the conclusion that *R *alone was not an appropriate factor of estimation. The fact that the factor *θ*_1 _grows consistently with *p *may have over-shadowed the importance of these factors.

## Discussion

The goal for this study was to produce an estimating function using basic network measurements. Specifically, we aimed to obtain a function such that only a single factor is used for estimation. In principle, it was possible to use a multivariate (polynomial) regression method using a number of the factors described in the section 'Identification of Significant Contributing Factors'. However, the independence assumption upon which this method is based was not applicable as the factors themselves tend to be highly correlated. Furthermore, it would have been difficult to interpret which factors were truly responsible for the increase in *p*, and would probably lead to inaccurate estimations in test models. Here, the most descriptive factor was *θ*_1_, which includes the clustering coefficients. The interpretation of the clustering coefficient used in this study is also often used in sociology and biochemical networks (see, for example, [15,17]). There are other interpretations of the clustering coefficient, such as that described by Soffer *et al*. [18]. Their definition eliminates degree-correlation biases, thus, quantifying the connectivity amongst the neighbors of a vertex independent to its degree and the degree of its neighbors. It would be interesting to use a similar definition for directed graphs and investigate its effects on ExPa estimation. Additionally, it is possible that other factors may provide a more accurate estimation for the number of ExPas. However, these factors may only be found by detailed analyses of network structures.

The estimating function given by Equation (3) typically estimated the number of ExPas of the test models to within a factor of 10. In cases where it failed, it did not under-estimate the number of ExPas. The version of the *E. coli *reconstructed network used by Klamt *et al*. [14] was not elementally- and charge-balanced and has since been replaced by updated versions [19,20]. We used a revised version *iJE660a*, which was found to be the closest to what they used, and is publicly available [19,21], to compare our method with Klamt's. When the estimating function was applied to this version, assuming that all reactions were active concurrently, 7 × 10^12 ^ExPas were estimated with our method, whereas Klamt's method yielded an upper-bound of 5 × 10^13 ^after disregarding inactive reactions in the unbalanced and smaller model. Given that *iJE660a *has 41 more reactions and all the reactions are elementally- and charge-balanced, we are confident that our estimating function can also serve as a conservative upper-bound of the number of ExPas after some adjustments. For larger networks such as the latest published reconstruction of *E. coli *consisting of 904 cited reactions [20], we estimate 3 × 10^18 ^ExPas, The Human reconstructed network with 3311 reactions [22] is predicted to have 10^29 ^ExPas when all reactions were active concurrently.

## Conclusion

In this study, we investigated the possibility of estimating with confidence the number of extreme pathways (ExPa), *p*, for metabolic networks. Our effort concentrated on the use of simple network measurements, namely the incoming and outgoing degrees, d∓i
 MathType@MTEF@5@5@+=feaafiart1ev1aaatCvAUfKttLearuWrP9MDH5MBPbIqV92AaeXatLxBI9gBaebbnrfifHhDYfgasaacH8akY=wiFfYdH8Gipec8Eeeu0xXdbba9frFj0=OqFfea0dXdd9vqai=hGuQ8kuc9pgc9s8qqaq=dirpe0xb9q8qiLsFr0=vr0=vr0dc8meaabaqaciaacaGaaeqabaqabeGadaaakeaacqWGKbazdaWgaaWcbaGaeS4eI02aaSbaaWqaaiabdMgaPbqabaaaleqaaaaa@30EF@ and the clustering coefficients, *c*_*i*_, for each of the active reactions, *r*_*i *_∈ *R*_*eff*_. In particular, it was found that log [*p*] was correlated to the factors θ1=∑i=1Rd−id+ici
 MathType@MTEF@5@5@+=feaafiart1ev1aaatCvAUfKttLearuWrP9MDH5MBPbIqV92AaeXatLxBI9gBaebbnrfifHhDYfgasaacH8akY=wiFfYdH8Gipec8Eeeu0xXdbba9frFj0=OqFfea0dXdd9vqai=hGuQ8kuc9pgc9s8qqaq=dirpe0xb9q8qiLsFr0=vr0=vr0dc8meaabaqaciaacaGaaeqabaqabeGadaaakeaaiiGacqWF4oqCdaWgaaWcbaGaeGymaedabeaakiabg2da9maaqadabaGaemizaq2aaSbaaSqaaiabgkHiTmaaBaaameaacqWGPbqAaeqaaaWcbeaakiabdsgaKnaaBaaaleaacqGHRaWkdaWgaaadbaGaemyAaKgabeaaaSqabaGccqWGJbWydaWgaaWcbaGaemyAaKgabeaaaeaacqWGPbqAcqGH9aqpcqaIXaqmaeaacqWGsbGua0GaeyyeIuoaaaa@41E3@ and θ2=∑i=1Rd−id+i
 MathType@MTEF@5@5@+=feaafiart1ev1aaatCvAUfKttLearuWrP9MDH5MBPbIqV92AaeXatLxBI9gBaebbnrfifHhDYfgasaacH8akY=wiFfYdH8Gipec8Eeeu0xXdbba9frFj0=OqFfea0dXdd9vqai=hGuQ8kuc9pgc9s8qqaq=dirpe0xb9q8qiLsFr0=vr0=vr0dc8meaabaqaciaacaGaaeqabaqabeGadaaakeaaiiGacqWF4oqCdaWgaaWcbaGaeGOmaidabeaakiabg2da9maaqadabaGaemizaq2aaSbaaSqaaiabgkHiTmaaBaaameaacqWGPbqAaeqaaaWcbeaakiabdsgaKnaaBaaaleaacqGHRaWkdaWgaaadbaGaemyAaKgabeaaaSqabaaabaGaemyAaKMaeyypa0JaeGymaedabaGaemOuaifaniabggHiLdaaaa@3F05@ with an exponential relationship. These factors can be calculated quickly and easily, and were found to increase in values consistently with *p*. The resulting estimating functions, in particular that given by Equation (3), typically estimated the number of ExPas to within a factor of 10. Therefore we are confident that these estimating functions are scalable and can be reliably applied to larger networks. These estimating functions will therefore enable researchers to decide whether ExPa analysis is an appropriate investigative tool for their objectives.

The set of extreme pathways is the convex basis used for biochemical characterization of the null-space of the stoichiometric matrix for a biochemical reaction network. ExPa analyses have typically been used to characterize phenotypic states of metabolic networks and identify network redundancy. Beyond these uses, the singular value decomposition of the extreme pathway matrix has been used to identify eigenpathways that are capable of characterizing phenotypic states of a system [23,24]. Nevertheless, applications such as these require ExPas to be calculated prior to any analysis. The number of ExPas is set to increase dramatically with network size and complexity. In particular, with the increase in details of metabolic network reconstructions and the emergence of reconstruction of global transcription/translation networks, new techniques for calculating and analyzing ExPas are much needed. Since the goal of systems biology is to study an organism as a whole, different types of biochemical networks will eventually be combined so that the system can be studied in its entirety. To over-come future computational challenges as well as being equipped with the necessary analytical techniques should become our immediate goal.

## Methods

### Basic Concepts and Notations

#### Hypergraph

We introduce some basic concepts and notations that will assist us in describing the measurements needed. We first note that a metabolic network can be described as a directed-hypergraph, where a node represents a metabolite and an edge a reaction. The stoichiometric matrix, **S**, can thus be seen as a node-edge incidence matrix. A directed-hypergraph ℋ
 MathType@MTEF@5@5@+=feaafiart1ev1aaatCvAUfKttLearuWrP9MDH5MBPbIqV92AaeXatLxBI9gBaebbnrfifHhDYfgasaacH8akY=wiFfYdH8Gipec8Eeeu0xXdbba9frFj0=OqFfea0dXdd9vqai=hGuQ8kuc9pgc9s8qqaq=dirpe0xb9q8qiLsFr0=vr0=vr0dc8meaabaqaciaacaGaaeqabaqabeGadaaakeaat0uy0HwzTfgDPnwy1egaryqtHrhAL1wy0L2yHvdaiqaaliab=Tqiibaa@376D@(*V*, *E*) consists of nodes (vertices) *v *∈ *V *and edges *e *∈ *E*. Let the matrix **S **be the node-edge incident matrix such that *s*_*i*, *j *_< 0 if node *v*_*i *_is at the tail of edge *e*_*j*_, *s*_*i*, *j *_> 0 if *v*_*i *_is at the head of *e*_*j*_, and *s*_*i*, *j *_= 0 if *e*_*j *_does not contain *v*_*i*_. We define the set of nodes *v *that are tails (heads) of edge *r *to be *T*(*e*) (*H*(*e*)). It can easily be seen that *T*(*e*) ∩ *H*(*e*) = ∅.

#### Reaction Adjacency and Neighbourhood Matrices **Â**, Δ^
 MathType@MTEF@5@5@+=feaafiart1ev1aaatCvAUfKttLearuWrP9MDH5MBPbIqV92AaeXatLxBI9gBaebbnrfifHhDYfgasaacH8akY=wiFfYdH8Gipec8Eeeu0xXdbba9frFj0=OqFfea0dXdd9vqai=hGuQ8kuc9pgc9s8qqaq=dirpe0xb9q8qiLsFr0=vr0=vr0dc8meaabaqaciaacaGaaeqabaqabeGadaaakeaaiiqacuWFuoargaqcaaaa@2E27@

The adjacency matrix contains information about whether one reaction 'goes into' another. Using the notation introduced in the 'Hypergraph' section, two reactions *r*_*i *_and *r*_*j *_are adjacent if *H*(*r*_*i*_) ∩ *T*(*r*_*j*_) ≠ ∅ or *H*(*r*_*j*_) ∩ *T*(*r*_*i*_) ≠ ∅, that is, the intersection of the set of outputs of reaction *r*_*i *_and the set of inputs for reaction *r*_*j *_is non-empty, or vice versa. In particular, we say *r*_*i *_'goes into' to *r*_*j *_if *H*(*r*_*i*_) ∩ *T*(*r*_*j*_) ≠ ∅. The adjacency matrix **Â **is constructed from the stoichiometric matrix **S **by partitioning **S **into two digitized components **Ŝ**_+ _and **Ŝ**_-_, where s^+i,j=1
 MathType@MTEF@5@5@+=feaafiart1ev1aaatCvAUfKttLearuWrP9MDH5MBPbIqV92AaeXatLxBI9gBaebbnrfifHhDYfgasaacH8akY=wiFfYdH8Gipec8Eeeu0xXdbba9frFj0=OqFfea0dXdd9vqai=hGuQ8kuc9pgc9s8qqaq=dirpe0xb9q8qiLsFr0=vr0=vr0dc8meaabaqaciaacaGaaeqabaqabeGadaaakeaacuWGZbWCgaqcamaaBaaaleaacqGHRaWkdaWgaaadbaGaemyAaKMaeiilaWIaemOAaOgabeaaaSqabaGccqGH9aqpcqaIXaqmaaa@3509@ if *s*_*i*, *j *_> 0 and s^−i,j=1
 MathType@MTEF@5@5@+=feaafiart1ev1aaatCvAUfKttLearuWrP9MDH5MBPbIqV92AaeXatLxBI9gBaebbnrfifHhDYfgasaacH8akY=wiFfYdH8Gipec8Eeeu0xXdbba9frFj0=OqFfea0dXdd9vqai=hGuQ8kuc9pgc9s8qqaq=dirpe0xb9q8qiLsFr0=vr0=vr0dc8meaabaqaciaacaGaaeqabaqabeGadaaakeaacuWGZbWCgaqcamaaBaaaleaacqGHsisldaWgaaadbaGaemyAaKMaeiilaWIaemOAaOgabeaaaSqabaGccqGH9aqpcqaIXaqmaaa@3514@ if *s*_*i*, *j *_< 0. Let **S **be an *n *× *m *matrix. The adjacency matrix for the reactions is then given by

A^=(a^i,j)∈Mn×n(0,1)such thata^i,j={1if i≠jands^+i⋅s^−j≠0,0otherwise.
 MathType@MTEF@5@5@+=feaafiart1ev1aaatCvAUfKttLearuWrP9MDH5MBPbIqV92AaeXatLxBI9gBaebbnrfifHhDYfgasaacH8akY=wiFfYdH8Gipec8Eeeu0xXdbba9frFj0=OqFfea0dXdd9vqai=hGuQ8kuc9pgc9s8qqaq=dirpe0xb9q8qiLsFr0=vr0=vr0dc8meaabaqaciaacaGaaeqabaqabeGadaaakeaafaqabeqadaaabaacbeGaf8xqaeKbaKaacqGH9aqpcqGGOaakcuWGHbqygaqcamaaBaaaleaacqWGPbqAcqGGSaalcqWGQbGAaeqaaOGaeiykaKIaeyicI4Saemyta00aaSbaaSqaaiabd6gaUjabgEna0kabd6gaUbqabaGccqGGOaakcqaIWaamcqGGSaalcqaIXaqmcqGGPaqkaeaacqqGZbWCcqqG1bqDcqqGJbWycqqGObaAcqqGGaaicqqG0baDcqqGObaAcqqGHbqycqqG0baDaeaacuWGHbqygaqcamaaBaaaleaacqWGPbqAcqGGSaalcqWGQbGAaeqaaOGaeyypa0ZaaiqabeaafaqaaeGaeaaaaeaacqaIXaqmaeaacqqGPbqAcqqGMbGzcqqGGaaicqWGPbqAcqGHGjsUcqWGQbGAaeaacqqGHbqycqqGUbGBcqqGKbazaeaacuWFZbWCgaqcamaaBaaaleaacqGHRaWkdaWgaaadbaGaemyAaKgabeaaaSqabaGccqGHflY1cuWFZbWCgaqcamaaBaaaleaacqGHsisldaWgaaadbaGaemOAaOgabeaaaSqabaGccqGHGjsUcqaIWaamcqGGSaalaeaacqaIWaamaeaacqqGVbWBcqqG0baDcqqGObaAcqqGLbqzcqqGYbGCcqqG3bWDcqqGPbqAcqqGZbWCcqqGLbqzaeaaaeaaaaaacaGL7baaaaGaeiOla4caaa@7E6B@

The elements of the adjacency matrix a^i,j
 MathType@MTEF@5@5@+=feaafiart1ev1aaatCvAUfKttLearuWrP9MDH5MBPbIqV92AaeXatLxBI9gBaebbnrfifHhDYfgasaacH8akY=wiFfYdH8Gipec8Eeeu0xXdbba9frFj0=OqFfea0dXdd9vqai=hGuQ8kuc9pgc9s8qqaq=dirpe0xb9q8qiLsFr0=vr0=vr0dc8meaabaqaciaacaGaaeqabaqabeGadaaakeaacuWGHbqygaqcamaaBaaaleaacqWGPbqAcqGGSaalcqWGQbGAaeqaaaaa@31CB@ = 1 if and only if there exists an metabolite *m*_*k *_such that *s*_*k*, *i *_> 0 and *s*_*k*, *j *_< 0; *i.e*. reaction *r*_*i *_'goes into' reaction *r*_*j*_. In terms of the matrix **Â**, two reactions *r*_*i *_and *r*_*j *_are adjacent if either a^i,j
 MathType@MTEF@5@5@+=feaafiart1ev1aaatCvAUfKttLearuWrP9MDH5MBPbIqV92AaeXatLxBI9gBaebbnrfifHhDYfgasaacH8akY=wiFfYdH8Gipec8Eeeu0xXdbba9frFj0=OqFfea0dXdd9vqai=hGuQ8kuc9pgc9s8qqaq=dirpe0xb9q8qiLsFr0=vr0=vr0dc8meaabaqaciaacaGaaeqabaqabeGadaaakeaacuWGHbqygaqcamaaBaaaleaacqWGPbqAcqGGSaalcqWGQbGAaeqaaaaa@31CB@ or a^j,i
 MathType@MTEF@5@5@+=feaafiart1ev1aaatCvAUfKttLearuWrP9MDH5MBPbIqV92AaeXatLxBI9gBaebbnrfifHhDYfgasaacH8akY=wiFfYdH8Gipec8Eeeu0xXdbba9frFj0=OqFfea0dXdd9vqai=hGuQ8kuc9pgc9s8qqaq=dirpe0xb9q8qiLsFr0=vr0=vr0dc8meaabaqaciaacaGaaeqabaqabeGadaaakeaacuWGHbqygaqcamaaBaaaleaacqWGQbGAcqGGSaalcqWGPbqAaeqaaaaa@31CB@ is non-zero, which is in agreement with the above definition.

Reactions *r*_*i *_and *r*_*j *_are said to be *connected *if any of *H*(*r*_*i*_) ∩ *T*(*r*_*j*_), *T*(*r*_*i*_) ∩ *H*(*r*_*i*_), *H*(*r*_*i*_) ∩ *H*(*r*_*j*_) or *T*(*r*_*i*_) ∩ *T*(*r*_*j*_) is non-empty. The neighbourhood matrix is given in the form

Δ^=(δ^i,j)such thatδ^i,j={1if i≠jands^i⋅s^j≠0,0otherwise,
 MathType@MTEF@5@5@+=feaafiart1ev1aaatCvAUfKttLearuWrP9MDH5MBPbIqV92AaeXatLxBI9gBaebbnrfifHhDYfgasaacH8akY=wiFfYdH8Gipec8Eeeu0xXdbba9frFj0=OqFfea0dXdd9vqai=hGuQ8kuc9pgc9s8qqaq=dirpe0xb9q8qiLsFr0=vr0=vr0dc8meaabaqaciaacaGaaeqabaqabeGadaaakeaafaqabeqadaaabaacceGaf8hLdqKbaKaacqGH9aqpdaqadaqaaGGaciqb+r7aKzaajaWaaSbaaSqaaiabdMgaPjabcYcaSiabdQgaQbqabaaakiaawIcacaGLPaaaaeaacqqGZbWCcqqG1bqDcqqGJbWycqqGObaAcqqGGaaicqqG0baDcqqGObaAcqqGHbqycqqG0baDaeaacuGF0oazgaqcamaaBaaaleaacqWGPbqAcqGGSaalcqWGQbGAaeqaaOGaeyypa0ZaaiqabeaafaqaaeGaeaaaaeaacqaIXaqmaeaacqqGPbqAcqqGMbGzcqqGGaaicqWGPbqAcqGHGjsUcqWGQbGAaeaacqqGHbqycqqGUbGBcqqGKbazaeaaieqacuqFZbWCgaqcamaaBaaaleaacqWGPbqAaeqaaOGaeyyXICTaf03CamNbaKaadaWgaaWcbaGaemOAaOgabeaakiabgcMi5kabicdaWiabcYcaSaqaaiabicdaWaqaaiabb+gaVjabbsha0jabbIgaOjabbwgaLjabbkhaYjabbEha3jabbMgaPjabbohaZjabbwgaLbqaaaqaaaaaaiaawUhaaiabcYcaSaaaaaa@70E5@

where s^i,j=1
 MathType@MTEF@5@5@+=feaafiart1ev1aaatCvAUfKttLearuWrP9MDH5MBPbIqV92AaeXatLxBI9gBaebbnrfifHhDYfgasaacH8akY=wiFfYdH8Gipec8Eeeu0xXdbba9frFj0=OqFfea0dXdd9vqai=hGuQ8kuc9pgc9s8qqaq=dirpe0xb9q8qiLsFr0=vr0=vr0dc8meaabaqaciaacaGaaeqabaqabeGadaaakeaacuWGZbWCgaqcamaaBaaaleaacqWGPbqAcqGGSaalcqWGQbGAaeqaaOGaeyypa0JaeGymaedaaa@33EF@ if *s*_*i*, *j *_is non-zero and 0 otherwise, and δ^i,j=1
 MathType@MTEF@5@5@+=feaafiart1ev1aaatCvAUfKttLearuWrP9MDH5MBPbIqV92AaeXatLxBI9gBaebbnrfifHhDYfgasaacH8akY=wiFfYdH8Gipec8Eeeu0xXdbba9frFj0=OqFfea0dXdd9vqai=hGuQ8kuc9pgc9s8qqaq=dirpe0xb9q8qiLsFr0=vr0=vr0dc8meaabaqaciaacaGaaeqabaqabeGadaaakeaaiiGacuWF0oazgaqcamaaBaaaleaacqWGPbqAcqGGSaalcqWGQbGAaeqaaOGaeyypa0JaeGymaedaaa@342C@ iff *r*_*i *_and *r*_*j *_are connected.

### Network Measurements

#### Effective Number of Reactions *R *= |*R*_*eff*_|

For any models of a reconstructed network, redundancy in terms of reactions that are not utilized is often expected. This is due to the fact that, for any specific model, there is a set of reactions that is not used under the specific simulation conditions, and therefore can be removed from the network without affecting the model's function. Extreme pathways are classified into 3 types, with Type-I being those that have exchange fluxes across the system boundaries that correspond to non-currency metabolites [25]. Here, we denote the set of reactions that are present in at least one Type-I ExPas by *R *= |*R*_*eff*_|. This number can be obtained by optimization techniques such as Flux-Balance Analysis [26] using tools such as SimPheny by Genomatica or FluxAnalyzer [27].

#### Reaction Connectivity *d*_±_(*r*_*i*_) = d±i
 MathType@MTEF@5@5@+=feaafiart1ev1aaatCvAUfKttLearuWrP9MDH5MBPbIqV92AaeXatLxBI9gBaebbnrfifHhDYfgasaacH8akY=wiFfYdH8Gipec8Eeeu0xXdbba9frFj0=OqFfea0dXdd9vqai=hGuQ8kuc9pgc9s8qqaq=dirpe0xb9q8qiLsFr0=vr0=vr0dc8meaabaqaciaacaGaaeqabaqabeGadaaakeaacqWGKbazdaWgaaWcbaGaeyySae7aaSbaaWqaaiabdMgaPbqabaaaleqaaaaa@31AA@

Having identified the set of reactions from a reconstruction that are active in a model, the stoichiometric matrix **S **can then be reduced by removing inactive reactions and metabolites. The connectivity (degree) of each active reaction can then be calculated. Since **S **can be considered the node-edge incidence matrix for a directed-hypergraph, it is more appropriate to consider the incoming and outgoing metabolites separately. The adjacency and connectivity between each pair of reactions can then be described in terms of the definition given in the section titled 'Reaction Adjacency and Neighbourhood Matrices **Â**, Δ^
 MathType@MTEF@5@5@+=feaafiart1ev1aaatCvAUfKttLearuWrP9MDH5MBPbIqV92AaeXatLxBI9gBaebbnrfifHhDYfgasaacH8akY=wiFfYdH8Gipec8Eeeu0xXdbba9frFj0=OqFfea0dXdd9vqai=hGuQ8kuc9pgc9s8qqaq=dirpe0xb9q8qiLsFr0=vr0=vr0dc8meaabaqaciaacaGaaeqabaqabeGadaaakeaaiiqacuWFuoargaqcaaaa@2E27@'. The number of reactions that produce at least one product which is consumed by reaction *r*_*i *_gives rise to the quantity d−i
 MathType@MTEF@5@5@+=feaafiart1ev1aaatCvAUfKttLearuWrP9MDH5MBPbIqV92AaeXatLxBI9gBaebbnrfifHhDYfgasaacH8akY=wiFfYdH8Gipec8Eeeu0xXdbba9frFj0=OqFfea0dXdd9vqai=hGuQ8kuc9pgc9s8qqaq=dirpe0xb9q8qiLsFr0=vr0=vr0dc8meaabaqaciaacaGaaeqabaqabeGadaaakeaacqWGKbazdaWgaaWcbaGaeyOeI0YaaSbaaWqaaiabdMgaPbqabaaaleqaaaaa@30A9@ = *d*_*i*_(*r*_*i*_), and the number of reactions that consume one or more of the products of *r*_*i *_is defined by the quantity d+i
 MathType@MTEF@5@5@+=feaafiart1ev1aaatCvAUfKttLearuWrP9MDH5MBPbIqV92AaeXatLxBI9gBaebbnrfifHhDYfgasaacH8akY=wiFfYdH8Gipec8Eeeu0xXdbba9frFj0=OqFfea0dXdd9vqai=hGuQ8kuc9pgc9s8qqaq=dirpe0xb9q8qiLsFr0=vr0=vr0dc8meaabaqaciaacaGaaeqabaqabeGadaaakeaacqWGKbazdaWgaaWcbaGaey4kaSYaaSbaaWqaaiabdMgaPbqabaaaleqaaaaa@309E@ = *d*_+_(*r*_*i*_).

**Figure 4:**
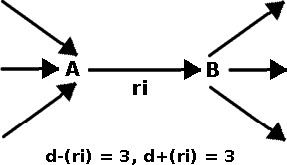
**Connectivity of Reactions**. Diagram describing different types of connectivities. Reaction *r*_*i *_utilizes metabolite *A*, which is produced by three reactions, and produces metabolite *B*, which is consumed by three reactions. Reaction *r*_*i *_then has an incoming degree of *d*_-_(*r*_*i*_) = 3 due to metabolite *A*, and outgoing degree of *d*_+_(*r*_*i*_) = 3.

From Figure 4, it can be seen that the number of possible pathways through a given reaction *r*_*i *_is given by d−i⋅d+i
 MathType@MTEF@5@5@+=feaafiart1ev1aaatCvAUfKttLearuWrP9MDH5MBPbIqV92AaeXatLxBI9gBaebbnrfifHhDYfgasaacH8akY=wiFfYdH8Gipec8Eeeu0xXdbba9frFj0=OqFfea0dXdd9vqai=hGuQ8kuc9pgc9s8qqaq=dirpe0xb9q8qiLsFr0=vr0=vr0dc8meaabaqaciaacaGaaeqabaqabeGadaaakeaacqWGKbazdaWgaaWcbaGaeyOeI0YaaSbaaWqaaiabdMgaPbqabaaaleqaaOGaeyyXICTaemizaq2aaSbaaSqaaiabgUcaRmaaBaaameaacqWGPbqAaeqaaaWcbeaaaaa@36EF@. It is tempting to conclude that the number of pathways calculated is given by ∏i=1Rd−i⋅d+i
MathType@MTEF@5@5@+=feaafiart1ev1aaatCvAUfKttLearuWrP9MDH5MBPbIqV92AaeXatLxBI9gBaebbnrfifHhDYfgasaacH8akY=wiFfYdH8Gipec8Eeeu0xXdbba9frFj0=OqFfea0dXdd9vqai=hGuQ8kuc9pgc9s8qqaq=dirpe0xb9q8qiLsFr0=vr0=vr0dc8meaabaqaciaacaGaaeqabaqabeGadaaakeaadaqeWaqaaiabdsgaKnaaBaaaleaacqGHsisldaWgaaadbaGaemyAaKgabeaaaSqabaGccqGHflY1cqWGKbazdaWgaaWcbaGaey4kaSYaaSbaaWqaaiabdMgaPbqabaaaleqaaaqaaiabdMgaPjabg2da9iabigdaXaqaaiabdkfasbqdcqGHpis1aaaa@3D53@. However, this would be similar to the number derived in [14], which typically over-estimated the number of elementary modes, of which the set of ExPas {**p**_*i*_} is a subset, by a factor of 6 × 10^7^. Here, we instead looked into the relationship between *p *= |{**p**_*i*_}| and the sum of these terms to avoid such an overestimation.

#### Reaction Clustering Coefficient *c*_*i *_= *c*(*r*_*i*_)

**Figure 5:**
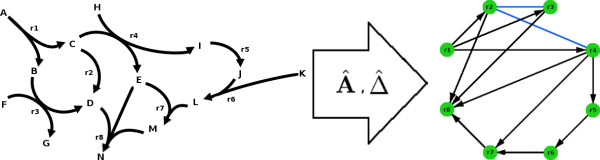
**Projection from Directed Hypergraph to One-mode Graph**. Projection from directed hypergraph to one-mode graph, where the hyperedges on the left-hand side become the nodes of the the graph on the right-hand side. A thick black arrow in the graph on the right signifies an edge *r*_*i *_is adjacent to *r*_*j*_, whereas a thin blue line signifies two edges connected that are not adjacent.

**Figure 6:**
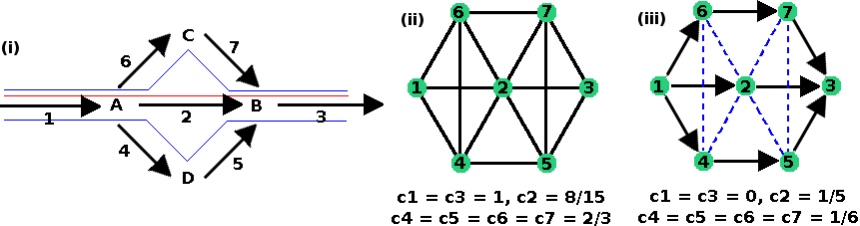
**Relationship between Reactions with Non-zero Clustering-coefficient and Alternative Routes**. Diagram showing relationship between non-zero clustering coefficients and alternative pathways. (i) shows three possible routes for a simple system; (ii) is the non-directed representation of this system using the above projection. The system has non-zero clustering coefficients, emphasizing alternative routes are possible; (iii) is the projection conforming to that shown in Figure 5, where non-zero clustering coefficient is found for 5 of the reactions that are involved in the branching points of alterative routes.

A metabolic network is described by the stoichiometric matrix **S**. This S-matrix can be seen as a node-edge incidence matrix for a directed hypergraph. However, the clustering coefficient for a hypergraph is not well defined. Since we are interested in how the reactions are connected, we can use the adjacency matrix, **Â**, which contains the node-node (reaction-reaction) information of the network, where a^i,j
 MathType@MTEF@5@5@+=feaafiart1ev1aaatCvAUfKttLearuWrP9MDH5MBPbIqV92AaeXatLxBI9gBaebbnrfifHhDYfgasaacH8akY=wiFfYdH8Gipec8Eeeu0xXdbba9frFj0=OqFfea0dXdd9vqai=hGuQ8kuc9pgc9s8qqaq=dirpe0xb9q8qiLsFr0=vr0=vr0dc8meaabaqaciaacaGaaeqabaqabeGadaaakeaacuWGHbqygaqcamaaBaaaleaacqWGPbqAcqGGSaalcqWGQbGAaeqaaaaa@31CB@ ≠ 0 if vertex *v*_*i *_is adjacent to *v*_*j*_, *i.e*., reaction *i *goes into reaction *j *(see Figure 5). In this configuration, we can then calculate the clustering coefficients for each active reaction using the usual equation

ci=c(ri)=|{ep,q}|ki(ki−1),
 MathType@MTEF@5@5@+=feaafiart1ev1aaatCvAUfKttLearuWrP9MDH5MBPbIqV92AaeXatLxBI9gBaebbnrfifHhDYfgasaacH8akY=wiFfYdH8Gipec8Eeeu0xXdbba9frFj0=OqFfea0dXdd9vqai=hGuQ8kuc9pgc9s8qqaq=dirpe0xb9q8qiLsFr0=vr0=vr0dc8meaabaqaciaacaGaaeqabaqabeGadaaakeaacqWGJbWydaWgaaWcbaGaemyAaKgabeaakiabg2da9iabdogaJnaabmaabaGaemOCai3aaSbaaSqaaiabdMgaPbqabaaakiaawIcacaGLPaaacqGH9aqpdaWcaaqaamaaemaabaWaaiWabeaacqWGLbqzdaWgaaWcbaGaemiCaaNaeiilaWIaemyCaehabeaaaOGaay5Eaiaaw2haaaGaay5bSlaawIa7aaqaaiabdUgaRnaaBaaaleaacqWGPbqAaeqaaOWaaeWaaeaacqWGRbWAdaWgaaWcbaGaemyAaKgabeaakiabgkHiTiabigdaXaGaayjkaiaawMcaaaaacqGGSaalaaa@4C35@

where *k*_*i *_is the number of reactions that *r*_*i *_is connected to, *i.e*., *k*_*i *_is the number of non-zero elements of the vector δ^i
 MathType@MTEF@5@5@+=feaafiart1ev1aaatCvAUfKttLearuWrP9MDH5MBPbIqV92AaeXatLxBI9gBaebbnrfifHhDYfgasaacH8akY=wiFfYdH8Gipec8Eeeu0xXdbba9frFj0=OqFfea0dXdd9vqai=hGuQ8kuc9pgc9s8qqaq=dirpe0xb9q8qiLsFr0=vr0=vr0dc8meaabaqaciaacaGaaeqabaqabeGadaaakeaaiiGacuWF0oazgaqcamaaBaaaleaacqWGPbqAaeqaaaaa@2FEF@ of the matrix Δ^
 MathType@MTEF@5@5@+=feaafiart1ev1aaatCvAUfKttLearuWrP9MDH5MBPbIqV92AaeXatLxBI9gBaebbnrfifHhDYfgasaacH8akY=wiFfYdH8Gipec8Eeeu0xXdbba9frFj0=OqFfea0dXdd9vqai=hGuQ8kuc9pgc9s8qqaq=dirpe0xb9q8qiLsFr0=vr0=vr0dc8meaabaqaciaacaGaaeqabaqabeGadaaakeaaiiqacuWFuoargaqcaaaa@2E27@. The set {*e*_*p*, *q*_} denotes the set of edges going from *r*_*p *_to *r*_*q *_with both *r*_*p *_and *r*_*q *_being connected to *r*_*i*_, *i.e*., *r*_*p *_and *r*_*q *_are connected to *r*_*i *_and are themselves adjacent. The denominator is the number of all such possible edges for a given *r*_*i*_. Note that since we are dealing with a directed graph, *e*_*p*, *q *_is not the same as *e*_*q*, *p*_. Alternative pathways from one set of substrates to one set of products almost always exist in biological networks, especially in metabolic networks. Figure 6 shows that non-zero clustering coefficients are related to alternative routes, and could then be an important factor for determining the number of extreme pathways calculated.

### Reconstructed Networks and Simulation Conditions

Reconstructed metabolic networks of *H. pylori *[3], Human Cardiac Mitochondria [4], the Human Red Blood Cell [5], and the core *E. coli *[28] network were used in this study. These networks are all mass- and charge-balanced, and were all tested on SimPheny (Genomatica) for the ability to produce biomass constituents. Furthermore, older versions of published, available networks that were used for extreme pathway and flux-balance analysis, which may not be completely balanced, were included in this study. This selection of networks represented a spectrum of complexity. Elementary network measurements, the number of generated models, and the source of each of these networks are detailed in Table 4.

**Table 4:** Basic Information of Models Used

**Network**	**# Rxns**	**Reversible**	**# Mets**	**Models Used**	**Source**
*H.pylori*	479	166	485	13	[3]
Mitochondria	200	92	238	16	[4]
Core *E.coli*	62	35	63	9	[28]
Central *E.coli*	56	19	62	2	[30]
Toy *E.coli*	50	37	53	4	[31]
Red Blood Cell	32	17	39	8	[5]

Simple measurements, statistics and sources of the models used in this study.

A total of 52 models were used from these networks. Most of these models were used to test for production of products given a specified substrate along with core exchange metabolites. The remaining 9 models included single-reaction deletions and/or the request for specific demand metabolites given a combination of primary substrates. The specific environmental conditions of these models are listed in Table 5, along with the names of abbreviated metabolites in Table 6.

**Table 5:** Environmental Conditions of Models. Table detailing the models used for estimation formulation, along with their environmental conditions.

	**Core Metabolites**		**Core Metabolites**				**Specification**
**Organisms**	**Name**	**Type**	**Inputs**	**Outputs**		**Free**	

**H. pylori**	co2	Free	Ac	ac	mal-L		Single Input along with core metabolite, allowing all outputs (unless specified as input only)
	fe2	Free	acac	akg	orn		
	fe3	Free	ad	asp-L	phe-L		
	h2o	Free	ade	acald	pro-L		
	h	Free	akg	etoh	pyr		
	nh4	Free	etoh	fum	ser-L		
	pi	Free	fum	glu-L	succ		
	so4	Free	gsn	gsn	thr-L		
	o2	Free	lac	gua	trp-L		
			mal	h2co3	tyr-L		
			pyr	hxan	urea		
			succ	lac-L			
			urea	lys-L			
**Mitochondria**	co2	Free	arachd	atp	glu-L		Single Input along with core metabolite, allowing all outputs (unless specified as input only)
	h	Free	bhb	pheme	gly		
	h2o	Free	crvnc	phs-L	glyc		
	fe2	Input	glc-D	12dgr_m	glyc3p		
	o2	Input	glu-L	acac	hdca		
	pi	Input	hdca	arachd	lac-L		
	urea	Output	lac-L	bhb	ocdca		
			ocdc All	coa	ocdcea		
			ocdca	crvnc	ocdcya		
			ocdcea	cys-L	pheme		
			ocdcya	glc-D	ps_m		
**Mitochondria**	co2, h, h20 fe2, fe3, o2 urea	Free	acac	atp		12dgr_m	(1) Single K/O of: CYOOm3, SUCD3-u10m
		Input	arachd	lac-L		coa	
		Output	Bhb	pheme		cys-L	
			crvnc	phs-L		glu-L	(2) Individual Request for: atp, phs-L, pheme
			glc-D			gly	
			glyc			ps_m	
			glyc3p				
			hdca				
			ocdca				
			ocdcea				
			ocdcya				
**E. coli Core**	co2	Free	ac	ac			Single Input along with core metabolite, allowing all outputs (unless specified as input only)
	h	Free	akg	akg			
	h2o	Free	etoh	etoh			
	o2	Input	for	for			
	pi	Input	fum	fum			
			glc-D	lac-D			
			lac-D	pyr			
			pyr	succ			
			succ				
**RBC**	adp, atp, co2, h, h2o, nad, nadh nadp, nadph, nh3, pi	Free	ade	23dpg	ino		(1) Single Input along with core metabolite, allowing all outputs (unless specified as input only)
		Free	ado	ade	lac		
		Free	glc	ado	pyr		
			hx	glc			
			ino	hx			
			lac				
			pyr				
			glc	23dpg		ade	(2) Multiple Inputs
				hx		ado	
						ino	
						pyr	
						ino	
**Central E. coli**	adp, atp, coa, co2, h, h2o, nad, nadh, nadp, nadph, pi, ppi	Free	glycogen	2dmmql8	mqn8		(1) Glycogen as Primary Input
		Free		2dmmq8	mql8		
		Free		3pg	oaa		
				akg	pep		
				amp	pyr		
				e4p	q8		
				fad	q8h2		
				fadh2	r5p		
			fad	2dmmq8	pyr		(2) A different set of Primary Input
			q8	fadh2	pep		
			2dmmql8	q8h2	e4p		
				amp	r5p		
				oaa	3pg		
				akg			

**Table 6:** External Metabolites Abbreviation

**Exchange Metabolite Abbreviation**			
12dgr_m	1,2-Diacylglycerol	hx	Hypoxanthine
23dpg	2,3-Phospho-D-glyceroyl phosphate	hxan	Hypoxanthine
2dmmq8	2-Demethylmenaquinone 8	ile-L	L-Isoleucine
2dmmql8	2-Demethylmenaquinol 8	ino	Inosine
3pg	3-Phospho-D-glycerate	lac	Lactate
ac	Acetate	lac-L	L-Lactate
acac	Acetoacetate	leu-L	L-Leucine
acald	Acetaldehyde	lys-L	L-Lysine
ad	Acetamide	mal	Malate
ade	Adenine	mal-L	L-Malate
ado	Adenosine	meoh	Methanol
adp	ADP	mql8	Menaquinol 8
akg	2-Oxoglutarate	mqn8	Menaquinone 8
ala-L	L-Alanine	nad	Nicotinamide adenine dinucleotide
ala-S	S-Alanine	nadh	Nicotinamide adenine dinucleotide – reduced
amp	AMP	nadp	Nicotinamide adenine dinucleotide phosphate
arachd	Arachidonic Acid (C20:4)	nadph	Nicotinamide adenine dinucleotide phosphate – reduced
asp-L	L-Asparagine	nh3	Ammonium
atp	ATP	nh4	Ammonium Ion
bhb	(2)-3-Hydroxybuanoate	o2	O2
ch4	Methan	oaa	Oxaloacetate
co2	CO2	ocdc All	octadecanoate, octadecenoate, octadecynoate
coa	Coenzyme A	ocdca	octadecanoate
crvnc	Cervonic Acid (C22:6, n-3)	ocdcea	octadecenoate
cys-L	L-Cysteine	ocdcya	octadecynoate
e4p	D-Erythrose 4-phosphate	orn	Ornithine
etoh	Ethanol	pep	Phosphoenolpyruvate
fad	Flavin adenine dinucleotide	phe-L	L-Phenylalanine
fadh2	Flavin adenine dinucleotide (reduced form)	pheme	Protoheme
fe2	Iron (II)	phs-L	Phospholipid
fe3	Iron (III)	pi	Phosphate
for	Formate	ppi	Diphosphate
fum	Fumarate	pro-L	L-Proline
glc	Glucose	ps_m	Phosphatidylserine
glc-D	D-Glucose	pyr	Pyruvate
glu-L	L-Glutamate	q8	Ubiquinone-8
gly	Glycine	q8h2	Ubiquinol-8
glyc	Glycerol	r5p	alpha-D-Ribose 5-phosphate
glyc3p	Glycerol 3-phosphate	ser-L	L-Serine
gsn	Guanosine	so4	Sulfate
gua	Guanine	succ	Succinate
h	H+	thr-L	L-Threonine
h2	H2	trp-L	L-Tryptophan
h2co3	carbonic acid	tyr-L	L-Tyrosine
h2o	H2O	urea	Urea
hdca	Hexadecanoate (n-C16:0)	val-L	L-Valine

Abbreviations of all external metabolite found in the 68 models used in this study.

A total of 16 functional models from 3 different organisms were used to test the validity of estimations formulated in the section 'Single Factor Estimate'. The networks used were 2 reduced versions of *H. pylori*, one consisting of 168 reactions and 170 metabolites, the other with 48 reactions and 65 metabolites. Reduced versions of the networks of *M. barkeri *[16] and *H. influenzae *[1] were also used. These consisted of 84 and 61 reactions and 121 and 83 metabolites, respectively. These networks are similar to the core *E. coli *model, with each reaction being mass- and charge-balanced. They were also tested for the production of biomass using SimPheny and hence are functional systems.

The number of data points may seem unorthodox. Although in theory it was possible to automatically generate networks of different input/output combinations, leading to a larger number of training and validation data points, in practice, only a few of such combinations would have resulted in models that produce non-Type-III extreme pathways as well as biomass constituents. For the validation stage, models producing biomass constituents as well as non-Type-III pathways that could be calculated quickly were desired. By drastically reducing networks, it was difficult to construct models that maintained biomass production.

In the case of *H. pylori*, three models were produced using the larger network in its entirety. Since the computation of ExPas is a time-consuming process, a smaller network was created to facilitate this process. This smaller network was subjected to random reaction-deletion while ensuring that the subsequent modified models could still produce equal amount of biomass. Five such models with random deletion were produced for this study. In addition, 5 models for *M. bakeri *and 3 for *H. influenzae *were generated in a similar fashion so that ExPa computation could be done within a reasonable computational time and effort. These are listed in Table 7.

**Table 7:** Environmental Conditions of Test Models

**Organism**	**#Rxn**	**#Met**	**External Metabolites**
*M. barkeri*	84	121	ac, ala-L, alac-S, ch4, co2, cys-L, gly, h, h2 h2o, ile-L, leu-L, meoh, pi, pyr, val-L
*H. influenzae*	61	83	ac, akg, co2, for, fum, glc-D, h, hxan nh4, mal-L, pi, pyr
*H. pylori*	48	65	acald, akg, co2, etoh, for, fum, glc-D, h h2co3, lac-L, mal-L, o2, pi
*H. pylori*	168	170	ac, acald, akg, asp-L, co2, etoh, fum, glc-D, glu-L, h, h2co3, h2o, lac-L, lys-L, mal-L, nh4, o2, phe-L, pi, pyr, ser-L, succ, thr-L, trp-L, tyr-L, urea

Simple network measurements and the lists of external metabolites for the networks used to test the capability of estimations developed in the section 'Single Factor Estimate'.

### Calculation of Extreme Pathways and Network Measurements

The extreme pathways of all models were computed using an implementation of the algorithm given in [29]. This implementation includes the C++ STL and the number theory library NTL. Algorithms for calculating the greatest common factor of a set of integers of arbitrary size and for sparse-matrix operations were also implemented. Network properties, including incoming and outgoing degrees and clustering coefficients of reactions, were calculated using a C++ implementation of the methods described in 'Basic Concepts and Notations' and 'Network Measurements' using sparse-matrix algorithms and bitwise operations.

### Correlation Coeffcients

Both Pearson's product-moment and Spearman's rank correlation coefficients were used as a guide to help identify important factors that contribute to the number of extreme pathways. The former is defined as

r=n∑i=1nxiyi−∑i=1nxi∑i=1nyin∑i=1nxi2−(∑i=1nxi)2n∑i=1nyi2−(∑i=1nyi)2
 MathType@MTEF@5@5@+=feaafiart1ev1aaatCvAUfKttLearuWrP9MDH5MBPbIqV92AaeXatLxBI9gBaebbnrfifHhDYfgasaacH8akY=wiFfYdH8Gipec8Eeeu0xXdbba9frFj0=OqFfea0dXdd9vqai=hGuQ8kuc9pgc9s8qqaq=dirpe0xb9q8qiLsFr0=vr0=vr0dc8meaabaqaciaacaGaaeqabaqabeGadaaakeaacqWGYbGCcqGH9aqpdaWcaaqaaiabd6gaUnaaqadabaGaemiEaG3aaSbaaSqaaiabdMgaPbqabaGccqWG5bqEdaWgaaWcbaGaemyAaKgabeaaaeaacqWGPbqAcqGH9aqpcqaIXaqmaeaacqWGUbGBa0GaeyyeIuoakiabgkHiTmaaqadabaGaemiEaG3aaSbaaSqaaiabdMgaPbqabaaabaGaemyAaKMaeyypa0JaeGymaedabaGaemOBa4ganiabggHiLdGcdaaeWaqaaiabdMha5naaBaaaleaacqWGPbqAaeqaaaqaaiabdMgaPjabg2da9iabigdaXaqaaiabd6gaUbqdcqGHris5aaGcbaWaaOaaaeaacqWGUbGBdaaeWaqaaiabdIha4naaDaaaleaacqWGPbqAaeaacqaIYaGmaaaabaGaemyAaKMaeyypa0JaeGymaedabaGaemOBa4ganiabggHiLdGccqGHsisldaqadaqaamaaqadabaGaemiEaG3aaSbaaSqaaiabdMgaPbqabaaabaGaemyAaKMaeyypa0JaeGymaedabaGaemOBa4ganiabggHiLdaakiaawIcacaGLPaaadaahaaWcbeqaaiabikdaYaaaaeqaaOWaaOaaaeaacqWGUbGBdaaeWaqaaiabdMha5naaDaaaleaacqWGPbqAaeaacqaIYaGmaaaabaGaemyAaKMaeyypa0JaeGymaedabaGaemOBa4ganiabggHiLdGccqGHsisldaqadaqaamaaqadabaGaemyEaK3aaSbaaSqaaiabdMgaPbqabaaabaGaemyAaKMaeyypa0JaeGymaedabaGaemOBa4ganiabggHiLdaakiaawIcacaGLPaaadaahaaWcbeqaaiabikdaYaaaaeqaaaaaaaa@8498@

for a series of *n *measurements *x*_*i *_and *y*_*i*_. It was used in this study as a guide to detect linear relationships amongst the data and estimating functions. The non-parametric correlation coefficient used in this study is defined as

ρ=1−6∑i=1nDi2n(n2−1),
 MathType@MTEF@5@5@+=feaafiart1ev1aaatCvAUfKttLearuWrP9MDH5MBPbIqV92AaeXatLxBI9gBaebbnrfifHhDYfgasaacH8akY=wiFfYdH8Gipec8Eeeu0xXdbba9frFj0=OqFfea0dXdd9vqai=hGuQ8kuc9pgc9s8qqaq=dirpe0xb9q8qiLsFr0=vr0=vr0dc8meaabaqaciaacaGaaeqabaqabeGadaaakeaaiiGacqWFbpGCcqGH9aqpcqaIXaqmcqGHsisldaWcaaqaaiabiAda2maaqadabaGaemiraq0aa0baaSqaaiabdMgaPbqaaiabikdaYaaaaeaacqWGPbqAcqGH9aqpcqaIXaqmaeaacqWGUbGBa0GaeyyeIuoaaOqaaiabd6gaUnaabmaabaGaemOBa42aaWbaaSqabeaacqaIYaGmaaGccqGHsislcqaIXaqmaiaawIcacaGLPaaaaaGaeiilaWcaaa@44DB@

where *D*_*i *_is the difference in the ranks of the corresponding values of the *n *pairs (*x*_*i*_, *y*_*i*_). This was used to decide whether a factor increased monotonically with the number of ExPas *p*.

## Authors' contributions

MY and BØP designed the study. MY performed and developed the programs used for the calculation of extreme pathways and network measurements, and analyzed and interpreted the data. IT provided all data of reconstructed models used in this study. MY drafted the manuscript while IT and BØP provided critical edits and important intellectual content. MY, IT & BØP have read and approved the final version of this manuscript.
